# Surveillance of tick-borne pathogens in ticks collected from Swiss residents

**DOI:** 10.1186/s13071-026-07363-8

**Published:** 2026-05-04

**Authors:** Ehsan Ghasemian, Bastian Marquis, Florian Tagini, Sébastien Aeby, Werner Tischhauser, Reto Lienhard, Christian Beuret, Virginie Martin, Silvan Hälg, Pie Müller, Antony Croxatto, Onya Opota, Gilbert Greub

**Affiliations:** 1 Institute of Microbiology, University Hospital Center and University of Lausanne, Rue du Bugnon 48, CH-1011 Lausanne, Switzerland; 2 Smartphone-App “Zecke–Tick Prevention”, ZHAW Spin-Off “A and K Strategy Ltd”, Neuhaus, SG Switzerland; 3 ADMED Microbiologie, Boucle de Cydalise 16, 2300 La Chaux-de-Fonds, Switzerland; 4 National Reference Center for Tick-Transmitted Diseases, Lausanne, La Chaux-de-Fonds, Switzerland; 5 Spiez Laboratory, Federal Office for Civil Protection, Spiez, Switzerland; 6 Swiss Tropical and Public Health Institute, Allschwil, Switzerland; 7 University of Basel, Basel, Switzerland

**Keywords:** Tick, Tick-borne disease, Prevalence, Spatial epidemiology, Switzerland, *Borrelia* spp., *Rickettsia* spp., *Chlamydiales*

## Abstract

**Background:**

Tick-borne diseases (TBDs) represent an increasing public health threat globally. Climate change has facilitated tick range expansion and extended active seasons, contributing to rising TBD incidence rates. In Switzerland, TBDs represent a major health concern. This study aims to characterise patterns in spatio-temporal distribution of tick-borne pathogens (TBPs) in ticks removed from humans across Switzerland, examine associations between tick developmental stages and TBP infection prevalence, and analyse co-infection patterns amongst different TBPs.

**Methods:**

We employed the “Tick Prevention” citizen science app to collect spatial and temporal data on tick bite incidents and to obtain tick specimens for pathogen screening throughout Switzerland during 2018–2020. Specimens underwent DNA extraction for TBP detection. Quantitative PCR targeted different TBPs at genus and species levels. Data analysis examined TBP infection prevalence in submitted ticks across geographic regions, seasons, and tick developmental stages, including co-infection patterns in ticks.

**Results:**

Of 1056 tick specimens, 352 (33.3%) tested positive for at least one TBP, with *Borrelia* spp. (16.3%) and *Rickettsia* spp. (12.69%) showing higher infection prevalence than other TBPs, including *Neoehrlichia mikurensis* (5%), *Anaplasma phagocytophilum* (1.8%), *Chlamydiales* (1.8%), and *Babesia* spp. (1.7%). Co-infections occurred in 59 specimens (5.6%), predominantly dual infections (5.2%), with *Borrelia* spp. and *N. mikurensis* representing the most common co-infection pattern. Among 58 larvae, 898 nymphs, and 96 adult ticks examined, tick infection prevalence increased with developmental stage, rising from larvae (18.9%) to nymphs (32.6%) to adults (48.9%), consistent with pathogen acquisition through successive blood meals during tick development. Spatially, TBPs were detected across 70 of 76 Swiss administrative regions, with most TBPs displaying uniform distribution. Temporally, tick-human encounters peaked during May–June (59.7% of announced events), with TBP detection rates remaining steady (28–37%) across the tick-active months from April to September. One-third of examined ticks harboured at least one TBP, with weighted models indicating infection prevalence in submitted ticks could reach 45% in certain Swiss Plateau regions.

**Conclusion:**

These findings emphasise the importance of continued tick and TBP surveillance programmes to inform public health interventions and prevention.

**Graphical Abstract:**

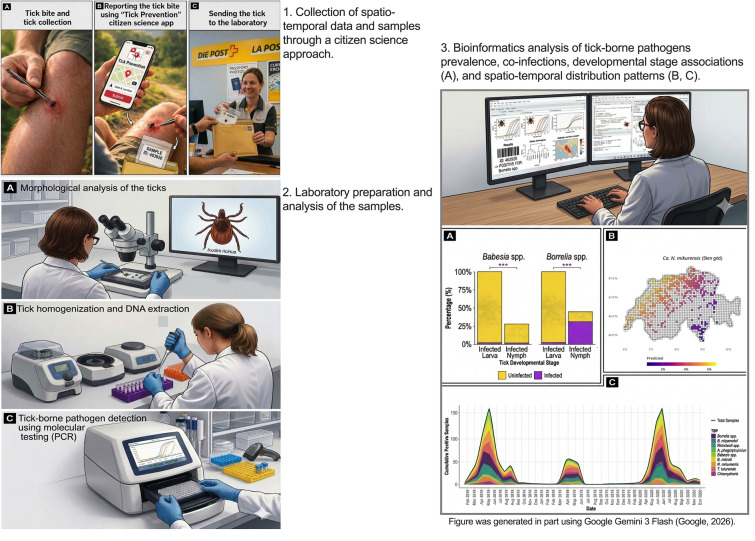

**Supplementary Information:**

The online version contains supplementary material available at 10.1186/s13071-026-07363-8.

## Background

Ticks are blood-feeding arthropods, ranging from 1 to 10 mm in size in the unfed state, that parasitise mammals, birds, reptiles, and occasionally amphibians [1]. In Europe, *Ixodes ricinus* is the predominant tick species responsible for human tick bites and serves as the primary vector for pathogenic microorganisms, including protozoa, bacteria, and viruses [2]. Tick-borne diseases (TBDs) are zoonotic infections that pose major threats to human and animal health worldwide [3–6].

Over 90% of bacterial species (spp.) transmitted by ticks belong to two orders, *Spirochaetales* and *Rickettsiales* [2, 7]. Lyme borreliosis, the most common TBD in Europe, is caused by members of the spirochete *Borrelia burgdorferi* sensu lato (s.l.) complex [7]. *Borrelia burgdorferi* s.l. is transmitted by *I. ricinus*, the most prevalent tick in Central Europe [8–11]. *Borrelia miyamotoi* is an exception among the tick-borne relapsing fever group, as it causes a mild febrile illness that is often subclinical, in contrast to other *Borrelia* species associated with relapsing fever [11, 12]. Tick-borne rickettsial diseases, including spotted fever group rickettsioses, constitute a diverse group of both emerging and established diseases caused by members of the order *Rickettsiales* [13]. In Europe, the most common species implicated in human disease include *Rickettsia helvetica*, *Rickettsia monacensis*, *Rickettsia conorii*, and *Rickettsia slovaca* [11, 14]. Infections with *R. helvetica* occasionally manifest as fever, headache, and myalgia [15]. Within the same order, the family *Anaplasmataceae* includes emerging pathogens in the genera *Anaplasma, Ehrlichia*, and *Neoehrlichia* [16]. The prevalence of *Anaplasma phagocytophilum* in *Ixodes* ticks varies across European countries, with an estimated prevalence of 1.7% in Switzerland [17–19]. *Anaplasma phagocytophilum* is the causative agent of human granulocytic anaplasmosis [17]. Another closely related bacterium, *Neoehrlichia mikurensis*, is an emerging pathogen in Europe and Asia that can occasionally cause life-threatening disease in immunocompromised individuals [20, 21]. Two other known bacterial TBDs include tularaemia and Q fever, caused by *Francisella tularensis* and *Coxiella burnetii*, respectively [22, 23].

Global warming has facilitated the emergence of new TBDs by enabling ticks to expand into previously unsuitable regions and encounter novel hosts. Milder winters and earlier springs have extended the active season of ticks and enhanced their reproductive cycles [24–27]. These climate changes have increased the risk of disease transmission, promoted the emergence of new TBDs, and posed greater public health challenges [24–27]. Changes in tick host distributions and ecosystem dynamics may further accelerate the spread of both established and emerging infections. Global warming enables ticks to push beyond both their latitudinal and altitudinal range limits [28]. An example comes from the Czech Republic's mountainous regions, where *I. ricinus* has expanded its range from 750 m to > 1000 m above sea level, coinciding with a 1.4 °C increase in mean annual air temperature over 2 decades [29]. Another example is Switzerland, where tick presence has been documented up to 1600 m elevation, and the area deemed suitable for ticks has grown from 16 to 25% of the total land area [30].

Switzerland is amongst the countries in which TBDs are a major public health concern [19, 31]. The “Tick Prevention” app, developed by the Zurich University of Applied Sciences, has served as an innovative citizen science tool designed to improve tick surveillance and enhance our understanding of TBD risks throughout Switzerland [32, 33]. The app enables users to report tick bite incidents with GPS-tagged data captured at 100-m spatial resolution, recording the location, date, and time of tick encounters. Users can voluntarily submit physical tick specimens to the Swiss National Reference Centre for Tick-transmitted Diseases (CNRT) for molecular pathogen screening. This crowd-sourced approach facilitates spatio-temporal analysis of tick encounter patterns and associated pathogen prevalence across the country [32, 33]. Given Switzerland's increasing incidence of TBD, this citizen science approach facilitates large-scale data collection that would be challenging to obtain through conventional field sampling alone [32].

Bald et al. [32] performed a comprehensive study assessing the spatio-temporal patterns of tick-human encounters in Switzerland from 2015 to 2021 using data collected through the “Tick Prevention” app. In this study, we complement their study by investigating the infection prevalence of tick-associated microorganisms in ticks submitted by citizens across Switzerland during 2018–2020 through the same citizen science initiative. We screened for both established and emerging TBPs, including *A. phagocytophilum*, *Babesia* spp., *B. microti*, *Borrelia* spp., *B. miyamotoi*, *Chlamydiales*, *C. burnetii*, *F. tularensis*, *N. mikurensis*, *Rickettsia* spp., and *R. helvetica*. Whilst we refer to these microorganisms collectively as TBPs throughout this article, we acknowledge that several taxa (members of the *Chlamydiales* order, *Rhabdochlamydia* spp., and *N. mikurensis*) represent emerging tick-borne bacteria with limited pathogenic evidence. Understanding co-infection patterns within tick vectors is important for assessing co-transmission risk and may reveal microbial interactions that influence pathogen establishment and vector competence [34–36]. In this study, we aim to (i) analyse the infection prevalence of different TBPs in submitted ticks and co-infection patterns amongst them, (ii) examine associations between tick developmental stages and TBP infection prevalence, and (iii) characterise the spatio-temporal distribution patterns of TBPs by integrating "Tick Prevention" app data with TBP detection results.

## Methods

### Tick and data collection

Tick specimens and associated data were obtained across Switzerland between February 2018 and December 2020 through a citizen science approach utilising the “Tick Prevention” app. This application, installed voluntarily by participants, serves a dual purpose: (i) providing users with information on high-risk areas and post-bite recommended protocols, whilst (ii) simultaneously collecting epidemiological data, including geographical coordinates of tick attachment to humans at a 100-m spatial resolution, to maintain updated risk assessment maps. In addition to contributing surveillance data, application users were offered the opportunity to submit collected tick specimens to the Swiss National Reference Centre for Tick-transmitted Diseases (CNRT) for downstream analysis. Participants submitted tick specimens by affixing individual ticks to a piece of paper using adhesive tape and recording the unique identification number generated by the app upon registration of the tick bite. Specimens were posted to the CNRT in standard envelopes at ambient temperature. Upon receipt at the laboratory, tick specimens were stored at room temperature until processing, which occurred monthly. This dry storage method has been validated for arthropod specimens, with studies demonstrating that room temperature storage for periods of up to several weeks preserves DNA integrity sufficiently for downstream molecular analyses [37–39]. Users were informed that these analyses were conducted for research purposes only and that they would not receive individual test results for the ticks they submitted.

### Sample preparation

Tick specimens were morphologically assessed via microscopic examination to determine their species, developmental stage, and (for adult ticks) sex. For TBP detection, individual ticks were mechanically homogenised in 600 μl of pre-cooled phosphate-buffered saline (PBS) using the TissueLyser system (Qiagen, Hilden, Germany). Following a brief centrifugation, 200 μl of the resulting supernatant was transferred to a Deepwell plate (Eppendorf, Hamburg, Germany) with or without 60 μl of glycerol for cryopreservation and stored at − 80 °C for subsequent analysis; 100 μl was used for DNA extraction.

### DNA extraction

DNA extraction was performed by combining 100 μl of tick homogenate supernatant with 400 μl of AVL buffer in a 96-well MagNA Pure processing cartridge (Qiagen, Hilden, Germany). Automated extraction was performed using the MagNA Pure 96 instrument with the MagNA Pure 96 DNA and Viral NA Large Volume kit following the Pathogen Universal LV 2.0 protocol.

### Polymerase chain reaction (PCR) and sequencing

Individual tick specimens were subjected to molecular analysis using quantitative PCR (qPCR) assays to detect TBPs following protocols established by Oechslin et al. [26] and Pilloux et al. [17]. Detection was performed at both genus and species levels, with genus-level screening targeting *Babesia* spp., *Borrelia* spp., and *Rickettsia* spp., and species-specific detection identifying *A. phagocytophilum*, *Babesia microti*, *B. miyamotoi*, *R. helvetica*, *N. mikurensis*, *F. tularensis*, and *C. burnetii*. Additionally, all samples were screened for members of the order *Chlamydiales* using pan-*Chlamydiales* PCR assays [40, 41]. TBPs were detected across all samples from 2018 to 2020, except for *C. burnetii*, tested only in 2019–2020 samples. Because *C. burnetii* was absent in over 62,889 Swiss ticks sampled by flagging in a separate study [17], we tested only a subset of samples for this pathogen. *Rickettsia helvetica* was the most prevalent rickettsial species detected when specifically screened in 2018. Accordingly, we considered the *Rickettsia* spp. PCR assay as an appropriate proxy for surveying *R. helvetica* in subsequent years.

The “*Borrelia* spp.” PCR detects all *Borrelia* spp., including *B. miyamotoi*. Therefore, the following decision criteria for the detection were used: a sample testing positive for *Borrelia* spp. PCR but negative for *B. miyamotoi* PCR was interpreted as positive for one or more species within the *Borrelia burgdorferi* s.l. complex. Conversely, a sample positive for both *Borrelia* spp. and *B. miyamotoi* was considered positive for *B. miyamotoi*, with the possibility of being also co-infected with a species belonging to the *B. burgdorferi* s.l. group.

Given the emerging significance of *Chlamydiales* as TBPs, the 2018 samples were additionally screened with a *Rhabdochlamydia*-specific PCR. PCR amplicons from 2018 samples exhibiting a cycle threshold (Ct) value < 35 in the pan-*Chlamydiales* assay were subjected to Sanger sequencing (Microsynth, Balgach, Switzerland). Taxonomic identification of resulting consensus sequences was done using the Basic Local Alignment Search Tool (BLAST) algorithm of the National Centre for Biotechnology Information (NCBI) GenBank database (https://www.ncbi.nlm.nih.gov/blast/) [42].

### Data analysis

All statistical analyses and visualisations were performed in R (v4.4.2) using RStudio (v2024.09.1) [43, 44]. Key packages included: dplyr, ggplot2, sf, tidyr, and spgwr [45–49]. Complete package details and versions are provided in Supplementary Methods. In addition, detailed statistical procedures and R code implementation are provided in Supplementary Methods.

### TBP infection prevalence

For each TBP, infection prevalence among submitted ticks was computed as the proportion of positive samples relative to total sample size, with 95% confidence intervals estimated using exact binomial methods. Co-infection patterns amongst TBPs were evaluated using Fisher's exact tests on pairwise 2 × 2 contingency tables and visualised using heat maps.

### TBP infection prevalence across tick developmental stages

Infection prevalence differences across developmental stages (larvae, nymphs, adults) were assessed using pairwise Fisher's exact tests ($\alpha$ = 0.05).

### Geospatial and regional analysis

Geographic coordinates from tick collection locations were spatially matched to Swiss postal code regions. Coordinates were validated for Swiss territorial boundaries and transformed to the Swiss national coordinate system (LV95, EPSG:2056). Population data for postal regions were obtained from the Swiss Federal Statistical Office (2024). Tick encounter rates were normalised per 10,000 inhabitants for each two-digit postal region. Spatial density patterns were visualised using kernel density estimation overlaid on topographic elevation data.

Elevation values were extracted from digital elevation model raster data and categorised into 200-m bins. Sample counts and TBP infection prevalence were calculated for each altitudinal stratum. Relationships between TBP infection prevalence and elevation were assessed using Pearson correlation and linear regression.

### Spatial analysis of TBP infection prevalence

Spatial patterns of TBP infection prevalence across Switzerland were analysed using grid-based Geographically Weighted Regression (GWR). Only TBPs with $\ge$ 10 positive samples were included. Tick sampling locations were aggregated into regular square grid cells, and infection prevalence was calculated for each grid cell. Optimal grid sizes (tested: 2, 5, 7, 10, 15, 20, 25, and 30 km) were determined for each TBP using a composite accuracy score incorporating: (i) Akaike information criterion (model fit), (ii) global R^2^ (explanatory power), (iii) cross-validation mean squared error (predictive accuracy), and (iv) Moran's I (spatial autocorrelation of residuals). The accuracy score assigned 30% weight to AIC and R^2^ each, and 20% to cross-validation error and spatial autocorrelation, prioritising model fit and explanatory power whilst maintaining predictive accuracy and spatial independence. GWR models used adaptive kernel bandwidth selected via AIC optimisation, with geographic coordinates as independent variables and TBP infection prevalence as the dependent variable.

Geographic variation in TBP infection prevalence across postal regions was evaluated using chi-square tests of independence or Fisher's exact tests (with Monte Carlo simulation when expected frequencies were < 5). Post hoc analyses compared each region against all others combined using Fisher's exact tests. For total TBP counts, regional differences were assessed using Kruskal-Wallis tests with Wilcoxon rank-sum post hoc comparisons. Poisson regression models were fitted as an alternative approach accounting for count data structure.

### Temporal analysis

Monthly tick-human encounter patterns were analysed using chi-square goodness-of-fit tests to assess uniform distribution across months. Individual month significance was determined using z-tests based on standardised residuals.

Monthly TBP infection prevalence was calculated with 95% confidence intervals (Wilson score method). Temporal patterns were assessed using chi-square tests or Fisher's exact tests (with Monte Carlo simulation when necessary). To account for variation in monthly tick abundance, standardised residual analysis compared observed versus expected monthly infection counts based on overall infection rates. For TBPs with $\ge$ 10 positive cases showing significant temporal patterns (*P* < 0.05), month-specific analyses identified peak periods, with post hoc pairwise comparisons using proportion tests and Bonferroni correction. Relationships between tick-human encounter intensity and TBP detection frequencies were quantified using Pearson correlation at the month-year level.

## Results

### Tick samples

A total of 1056 tick specimens were validated for analysis from the 1254 samples initially received; 198 samples were excluded because of incomplete data linkage between PCR results and corresponding sample identification records in the application database. Collection date information was available for all specimens, whilst geographic data (coordinates) were available for 1035 specimens. Developmental stage classification was determined for 1052 specimens (99.6%), comprising 898 nymphs (85.4%), 96 adults (9.1%), and 58 larvae (5.5%). Amongst the 96 adult specimens, sex determination revealed a predominance of females (*n* = 93, 96.9%) over males (*n* = 3, 3.1%). All specimens at nymph and adult stages were morphologically identified as *I. ricinus* based on established taxonomic characteristics [50].

### TBP infection prevalence and co-infections

**Fig. 1:**
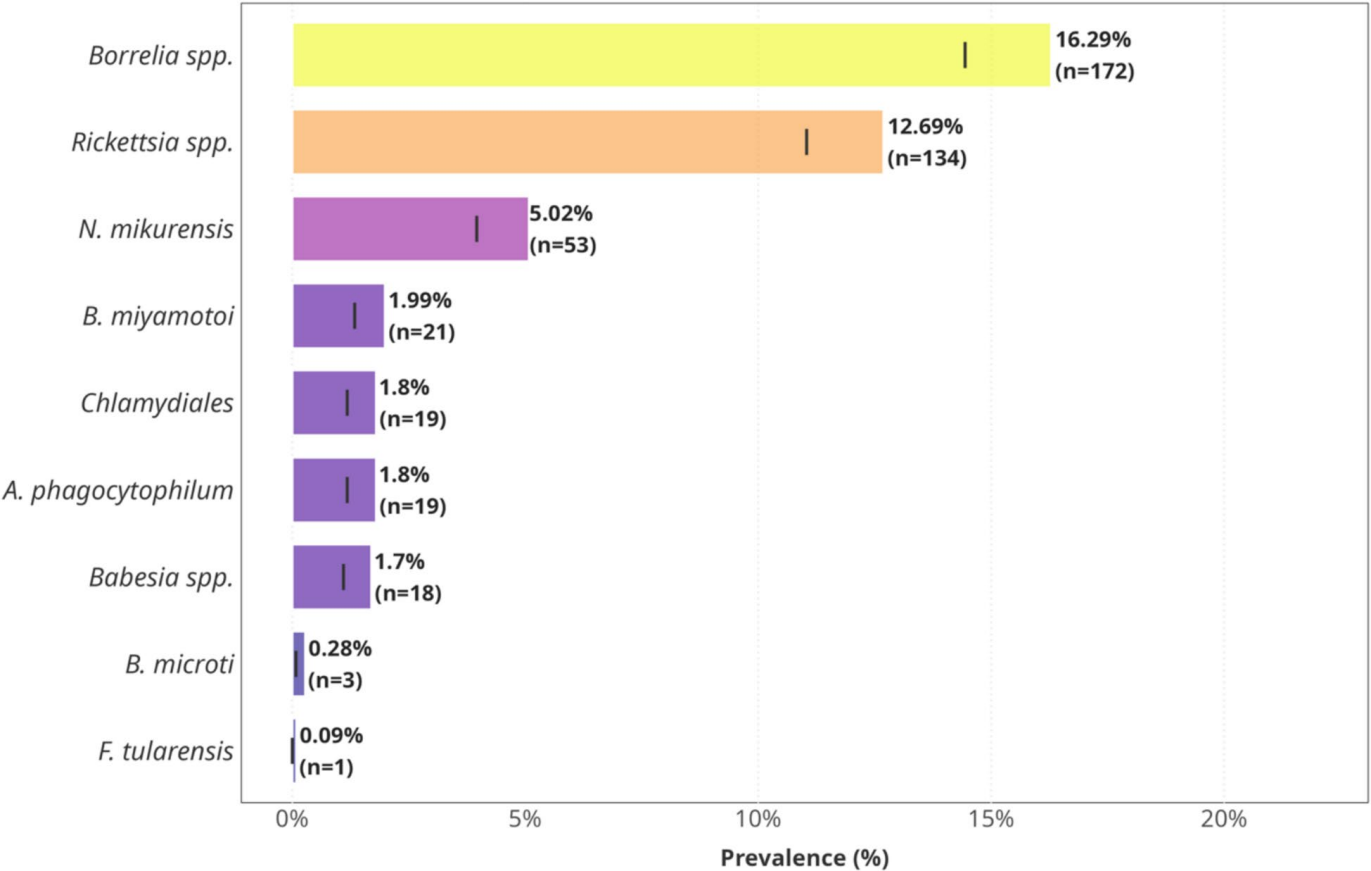
Prevalence of TBPs in tick samples. Horizontal bars represent the prevalence percentage for each TBP, ranked in descending order from highest to lowest prevalence. Error bars indicate 95% confidence intervals calculated using exact binomial methods. Sample counts (*n*) are displayed on each bar alongside prevalence percentages. *Coxiella burnetii* and *Rickettsia helvetica* are excluded from the analysis because of temporal limitations in screening protocols (see Methods section for details)

Overall, 33.3% (95% CI 30.5–36.2) of tick specimens tested positive for at least one TBP. TBP infection prevalence in submitted ticks varied considerably across pathogens, with two dominant genera detected: *Borrelia* spp. (16.3%, 95% CI 14.1–18.6) and *Rickettsia* spp. (12.7%, 95% CI 10.7–14.8) (Fig. 1). The remaining TBPs demonstrated lower infection prevalence in submitted ticks: *N. mikurensis* (5.0%, 95% CI 3.8–6.3), *B. miyamotoi* (1.99%, 95% CI 1.2–2.8), *A. phagocytophilum* (1.8%, 95% CI 1.0–2.7), *Chlamydiales* (1.8%, 95% CI 1.0–2.7), *Babesia* spp. (1.7%, 95% CI 0.9–2.6), *B. microti* (0.3%, 95% CI 0.0–0.7), and *F. tularensis* (0.1%, 95% CI 0.0–0.3) (Fig. 1). In 2018, *R. helvetica* was detected in 47 of 446 samples (10.5%, 95% CI 7.8–13.5). No specimens tested positive for *C. burnetii*.

Co-infection analysis revealed that most infected ticks harboured a single infection (27.7% of total specimens), whereas multiple TBP infections occurred in 5.6%. Dual infections represented most co-infections (5.2%), with less triple infections occurring less frequently (0.3%) and a single quadruple infection. The most prevalent dual co-infection was *Borrelia* spp. and *N. mikurensis* (*n* = 25, *P* < 0.001), followed by *Borrelia* spp. with *Rickettsia* spp. (*n* = 12, *P* = 0.019) (Table S1, Figure S1).

*Chlamydiales* were detected in 1.8% of the specimens, with a minor propensity for co-infection with *A. phagocytophilum* (*P* = 0.044) (Fig. 1, S1). Molecular analysis of the 2018 specimen subset (*n* = 446) revealed four testing positive for *Rhabdochlamydia* spp. Furthermore, 16S rRNA gene sequencing coupled with BLAST nucleotide database analysis identified three *Chlamydiales*-positive specimens from 2018 with the highest similarity to members of the *Parachlamydiaceae* family.

### TBP infection prevalence across developmental stages

Ticks comprised predominantly nymphs (85.4%), followed by adults (9.1%) and larvae (5.5%) (*P* < 0.0001). Overall, TBP infection prevalence in submitted ticks demonstrated an ontogenetic progression, with infection rates increasing across developmental stages: larvae (18.9%), nymphs (32.6%), and adults (48.9%). Pairwise comparisons revealed significant differences between all developmental stages (adults vs. larvae: *P* < 0.001; adults vs. nymphs: *P* < 0.01; nymphs vs. larvae: *P* = 0.03).

**Fig. 2:**
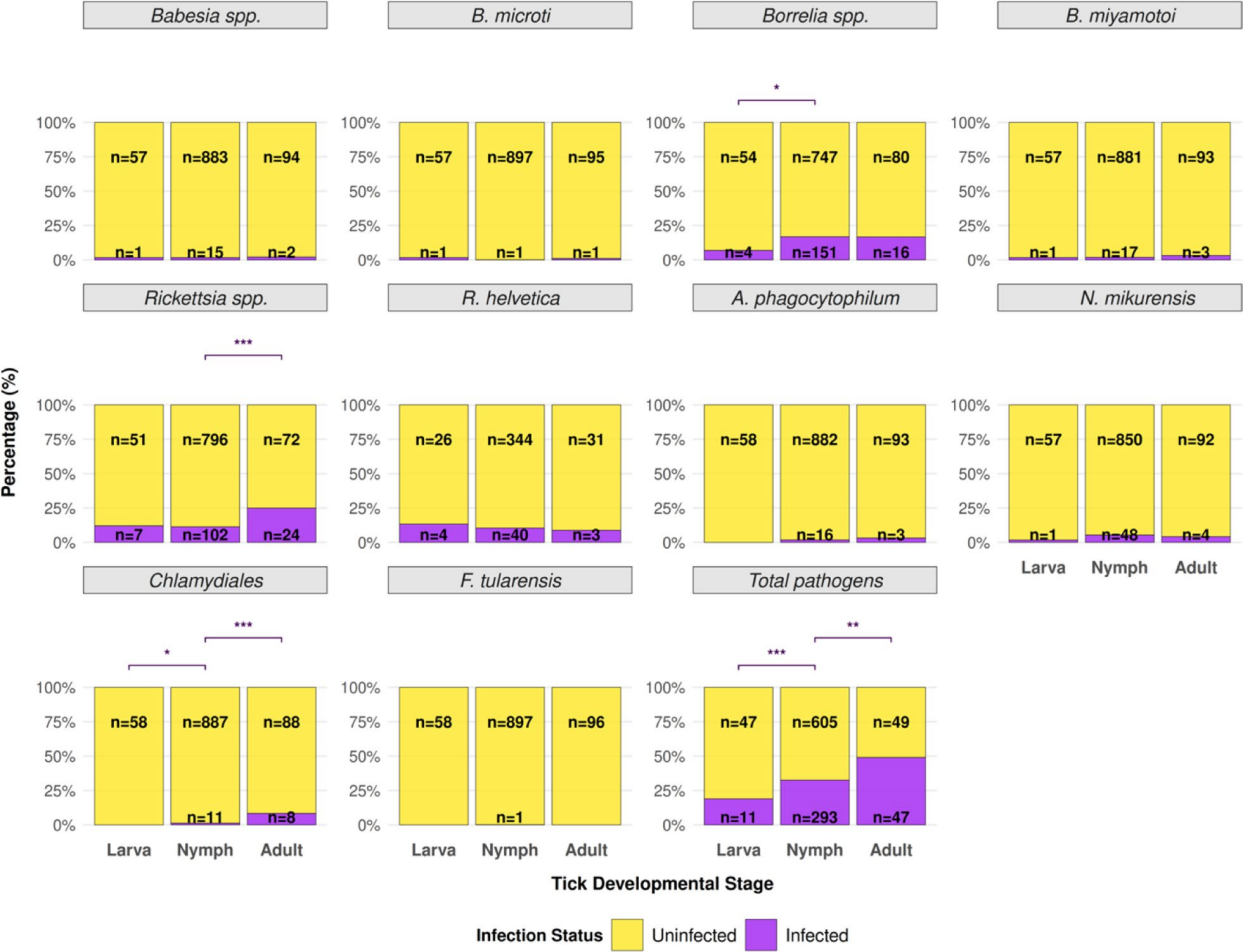
TBP prevalence across tick developmental stages. Each panel presents the infection status distribution for a specific TBP across three developmental stages (larva, nymph, adult). Stacked bars represent 100% of the tested tick population for each stage, with infected portions shown in purple and uninfected portions in yellow. Sample size annotations indicate actual counts: numbers at the bottom of bars show infected tick counts, whilst numbers at 75% height show uninfected tick counts. Asterisks denote statistically significant pairwise comparisons between developmental stages from Fisher's exact tests (* *P* < 0.05, ** *P* < 0.01, *** *P* < 0.001), with connecting brackets indicating which specific stage pairs differ significantly. *Rickettsia helvetica* data are based exclusively on 2018 specimens because of temporal screening limitations (see Methods section for details)

*Rickettsia* spp. and *Chlamydiales* showed significantly elevated infection prevalence in adults compared with nymphs (*Rickettsia* spp.: 25.0% vs. 11.4%, *P* < 0.001; *Chlamydiales*: 8.3% vs. 1.2%, *P* < 0.001) (Table S2, Fig. 2). *Borrelia* spp. demonstrated significantly higher infection rates in nymphs relative to larvae (16.8% vs. 0.1%, *P* = 0.04). The remaining TBPs showed no significant developmental stage-related variation (Table S2, Fig. 2).

### Geographic distribution of the ticks and TBPs

**Fig. 3:**
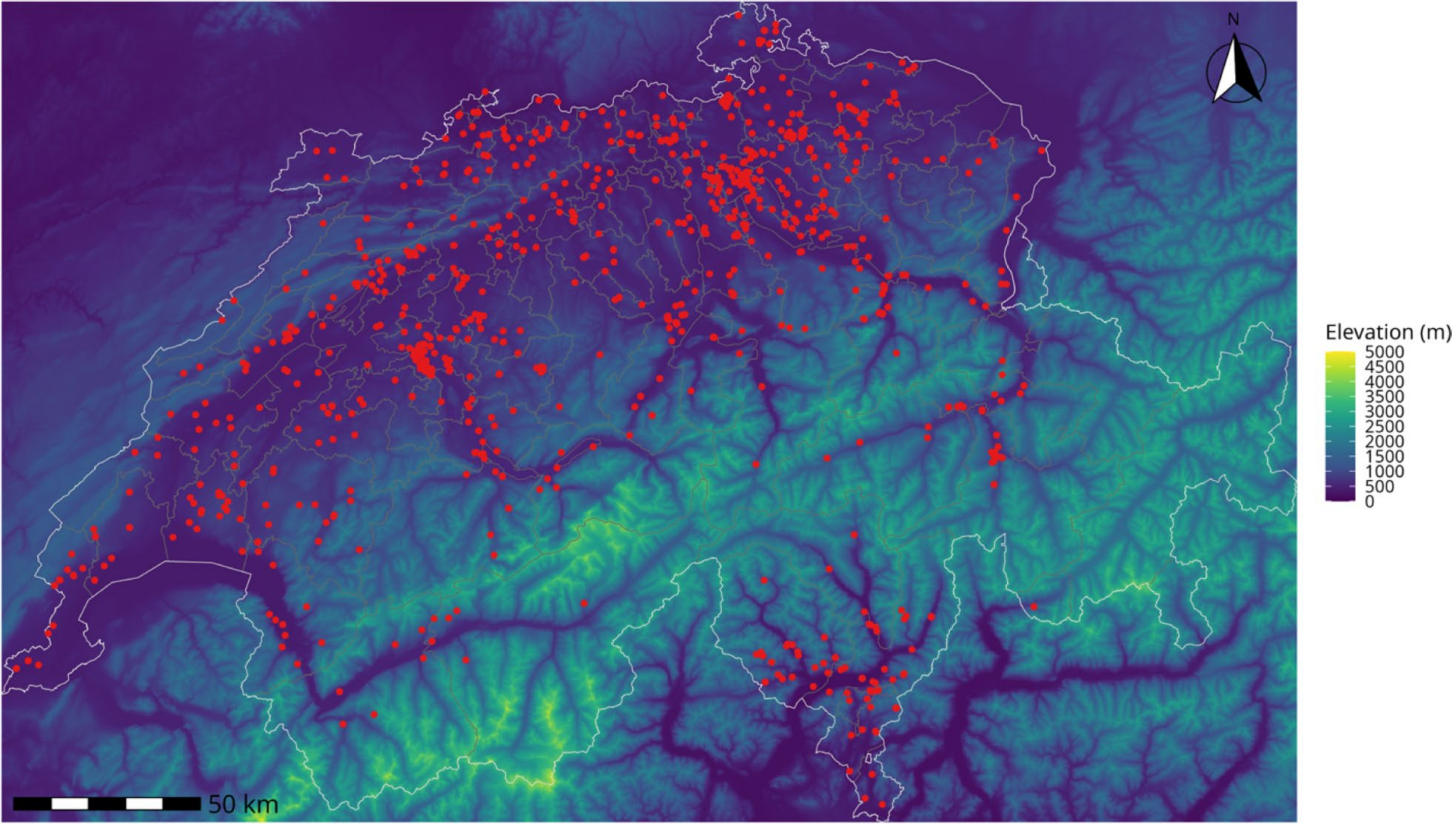
Spatial distribution of sample locations across Switzerland with topographic context and density analysis. The map displays elevation gradients using a colour-coded scale, with individual sample locations marked as red points. The white outline shows Switzerland's national border. A scale bar and north arrow provide geographic reference. (Data source: GPS coordinates & elevation data: AWS Terrain Titles)

The analysis of ticks received across Swiss postal regions revealed spatial heterogeneity. Across 76 two-digit postal regions analysed (Figure S2, Table S3), the overall national rate was 1.2 tick bite reports per 10,000 inhabitants. However, regional rates varied substantially, ranging from 0.1 to 8.5 tick bite reports per 10,000 inhabitants. The distribution of ticks received across Switzerland revealed spatial clustering, with samples concentrated in populated urban centers and at lower elevations (Fig. 3**, **S2).

TBPs were detected in 70 of 76 Swiss two-digit postal regions, with regional infection prevalence in submitted ticks ranging from 8.3% to 100% (mean sample size per positive region: 14.6 ± 12.7 ticks) (Table S3). Analysis of TBP distribution across Swiss two-digit postal regions revealed a positive correlation between the number of tick samples collected per region and the total number of TBP detections (*P* < 0.001) (Figure S3).

Screening of tick samples from 76 Swiss two-digit postal regions for TBPs revealed most TBPs, including the most prevalent *Borrelia* spp. (16.3%), displayed a non-significant deviation from a uniform distribution across Switzerland (Table S4), whilst only *Rickettsia* spp. demonstrated significant geographic clustering (*P* = 0.013). Postal region 86 (Region Dübendorf, Zürcher Oberland) emerged as a region with *Rickettsia* spp. infection prevalence in submitted ticks 5.47 times higher than in other regions (95% CI 1.99–14.47) (Table S4). In contrast to individual TBP clustering patterns, analysis of cumulative TBP infection prevalence in submitted ticks revealed no significant regional variation (*P* = 0.28) with no postal regions demonstrating significantly different TBP detection compared to the national average (Table S4).

**Fig. 4:**
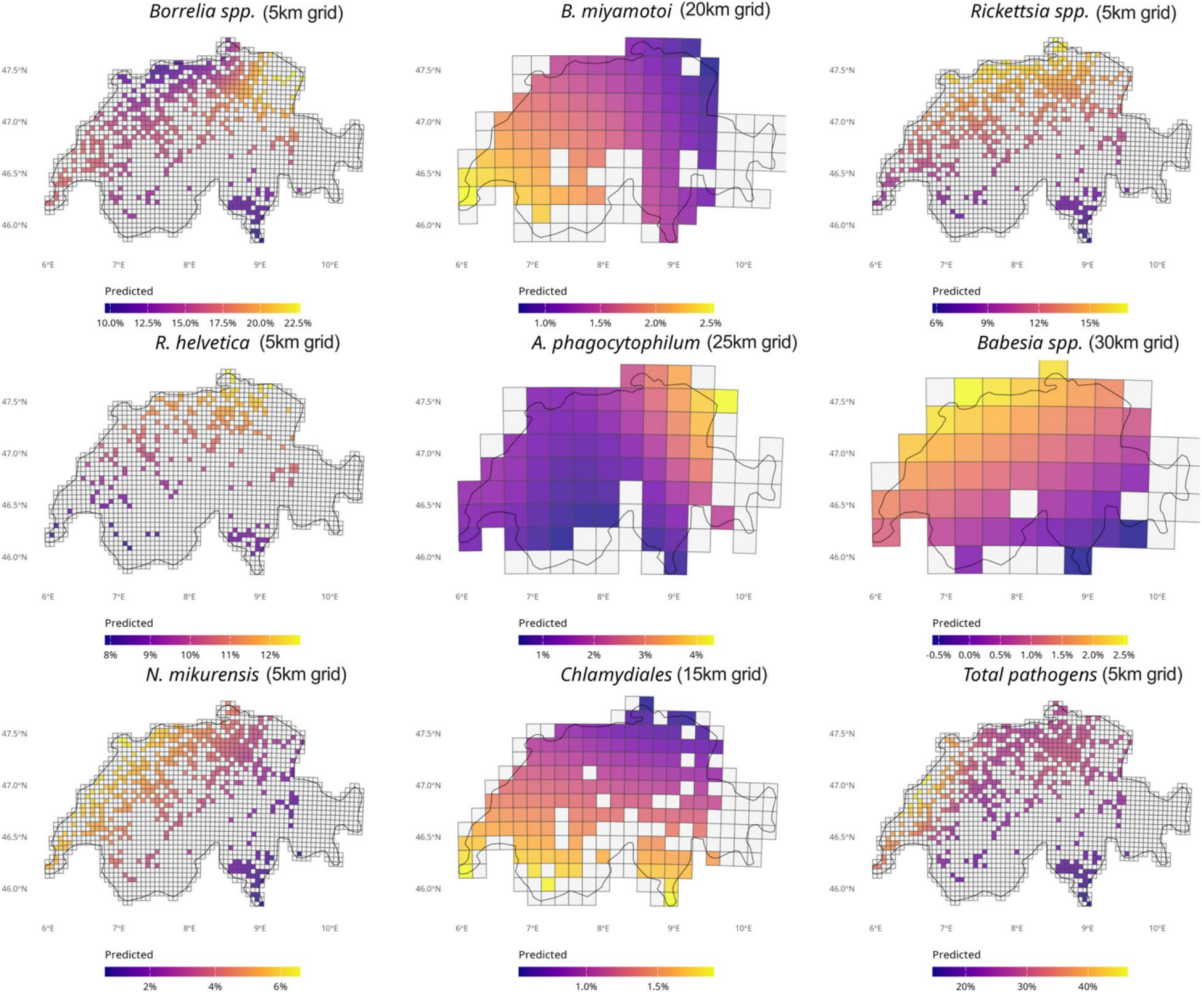
Predicted TBP prevalence across Switzerland using GWR models. Spatial predictions of TBP prevalence across Switzerland generated from TBP-specific GWR models. Each subplot shows model predictions for a TBP, using individually optimised grid size and adaptive bandwidth selection. Grid cells are coloured according to GWR-predicted prevalence values, representing the spatially varying regression model's estimates of TBP occurrence probability based on geographic coordinates. The predicted surfaces smooth the observed prevalence data whilst capturing local spatial trends and identifying areas of elevated or reduced TBP risk. Prediction accuracy varies spatially as indicated by local R^2^ values (Figure S2). The colour scale matches Figure S4 for direct comparison between observed and predicted prevalence patterns. Only TBPs with ≥ 10 positive samples across the entire dataset were included to ensure sufficient statistical power for reliable spatial modeling. Grid resolution: Each TBP uses its individually optimised grid cell size (ranging from 5–30 km) determined by grid size optimisation. Missing data: Grey areas indicate grid cells with insufficient data for analysis (fewer than required minimum samples). *Rickettsia helvetica* predictions are based on 2018 specimens because of temporal screening limitations (see Methods section for details)

The GWR models demonstrated heterogeneous predictive performance across different TBPs and geographic regions, as indicated by the Local R^2^ values (Figure S4). Residual analysis revealed systematic over- and under-prediction patterns, with some geographic areas consistently showing poor model fit across multiple TBPs (Figure S5). The total TBP burden model showed moderate spatial prediction accuracy, with better performance in regions with higher sampling density. Spatial predictions of infection prevalence in submitted ticks revealed distinct regional patterns: *Borrelia* spp. exhibited the highest predicted infection prevalence (20–22.5%) in northeastern Switzerland, whilst *Rickettsia* spp. infection prevalence peaked (20%) in northern regions. *Neoehrlichia mikurensis* showed elevated infection prevalence (6%) in western Switzerland compared to other regions (Fig. 4, S6). Cumulative TBP burden predictions indicated that 40–45% of submitted ticks in western and 30–35% in northeastern regions of the Swiss Plateau are likely to harbour at least one TBP (Fig. 4, S6).

Tick-human encounters were concentrated at lower elevations, with 50.8% of specimens (*n* = 1009) collected between 400 and 599 m, declining sharply to minimal encounters above 1400 m elevation (Figure S7). Linear regression analysis revealed no statistically significant associations between altitude and any individual TBP or total TBP burden (*P* > 0.05) (Table S5). However, some statistical trends can be observed: most TBPs, including *Borrelia* spp., *Rickettsia* spp., *N. mikurensis*, and total TBP burden, showed decreasing infection prevalence in submitted ticks with increasing elevation (Figure S8, Table S5). *Anaplasma phagocytophilum* infection prevalence showed an upward trend with elevation, though this was not significant (*P* = 0.069) (Figure S8, Table S5). Small sample sizes at elevations > 1000 m (*n* = 1–45 per bin) likely limited the statistical power to detect significant elevational patterns.

### Temporal distribution of the ticks and TBPs

**Fig. 5:**
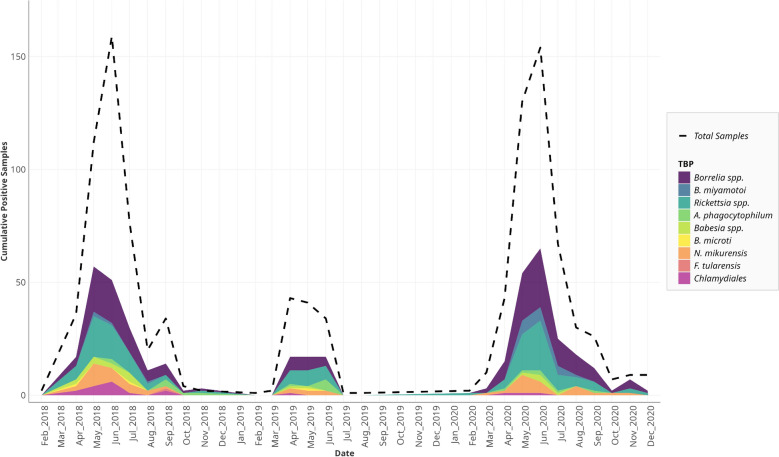
Temporal distribution of TBP detections, 2018–2020. Coloured stacked areas represent the cumulative contribution of each TBP to total positive detections per month, with different colours corresponding to nine analysed TBPs. The height of each coloured area indicates the number of positive detections for that specific TBP, whilst the total height represents the cumulative TBP burden across all species. The black dashed line overlays total sample collection numbers as a reference for samples received over the study period. *Coxiella burnetii* and *Rickettsia helvetica* are excluded from this analysis because of temporal limitations in screening protocols (see Methods section for details)

All temporal analyses were based on the date of tick-human encounter as recorded through the mobile application. Of the 1056 tick specimens, 42.3% were from 2018, 11.7% from 2019, and 46.1% from 2020. Temporal analysis of tick-human encounters revealed a pronounced seasonal pattern across 2018–2020 (Fig. 5). Peak encounters occurred during late spring and early summer, with June recording the highest frequency (32.8%, *P* < 0.001), followed by May (26.8%, *P* < 0.001), collectively representing 59.7% of all submissions. Encounter rates remained significantly elevated in July (13.7%, *P* < 0.001) and April (11.6%, *P* = 0.008). Moderate submission frequencies were observed during the transition months of August (4.8%) and September (5.7%), whilst winter and early spring months showed a substantially reduced activity: March (1.1%), February (0.5%), October (1.0%), November (1.0%), and December (0.8%) (Fig. 5).

Monthly TBP infection rates remained relatively stable across the active season (28.3–37.2%, April to September), indicating consistent TBP infection prevalence in submitted ticks regardless of encounter frequency (Table S6). Peak absolute TBP detections occurred during months of highest tick activity: June (36% infection rate), May (31.7% infection rate), and April (32.8% infection rate), which collectively accounted for 74.7% of all TBP detections (Fig. 5, Table S6). Compared with an expected uniform monthly distribution, May and June showed significantly higher absolute TBP detections (*P* < 0.001), whilst the winter months (January, February, October, November, December) had significantly lower detection counts (*P* < 0.001). November showed an elevated infection rate (72.7%, *P* = 0.006) compared to other months, though this finding was based on a limited sample size (*n* = 11).

When analysing TBP infection prevalence in submitted ticks adjusted for monthly submission volumes, there was a significant seasonal pattern (*P* = 0.016) for *A. phagocytophilum* with a distinctive bimodal distribution characterised by peaks during late spring and early autumn, separated by reduced infection prevalence during summer months (Table S6). September showed significantly elevated prevalence of *A. phagocytophilum* infection in submitted ticks (6.7%, *P* < 0.01). The total number of *A. phagocytophilum*-positive samples was limited (*n* = 19), and this finding should be interpreted with caution given the small sample size (Figure S10, Table S6).

## Discussion

Previously, Oechslin et al. [19] tested 1079 ticks collected from (sub-) urban areas of Switzerland between 2015 and 2016 for a comparable panel of TBPs. Their study revealed that 33.2% of ticks harboured at least one TBP and 6.6% carried multiple TBPs, findings that closely mirror our results. Similarly, a study from Finland reported that 30% of *I. ricinus* ticks were infected with at least one TBP [51].

Notably, about half of all *N. mikurensis* infections co-occurred with *Borrelia* spp., demonstrating a statistically significant association between these TBPs. *Neoehrlichia mikurensis* represents an emerging TBP in Europe, with several studies reporting infection prevalence rates of 3–10% in *Ixodes* ticks, consistent with our findings [20, 21, 52–55]. Supporting our observations, a study on ticks from southern Sweden reported that 46% of *N. mikurensis*-infected specimens were co-infected with *Borrelia* spp. [56]. Subsequently, multi-country European studies demonstrated an average four-fold higher likelihood of co-infection between *N. mikurensis* and *Borrelia afzelii* compared to other TBPs [18]. These observations suggest a potential mutualistic interaction between these bacteria during co-infection within tick vectors.

The increase in TBP infection prevalence in submitted ticks from larvae to nymphs to adults may suggest that ticks accumulate TBPs after each blood meal and throughout their developmental cycle [57–59], consistent with other findings from Switzerland, Australia, and Finland [60–62]. For *Borrelia* spp., nymphs exhibited higher infection prevalence than larvae, supporting earlier observations by Waindok et al. [63] and Hildebrandt et al. [64]. Similarly, *Rickettsia* spp. infection prevalence was elevated in adults compared to nymphs, supporting previous findings [65–67], although some studies have reported consistent *Rickettsia* spp. infection prevalence across all developmental stages [68–70]. The higher infection prevalence of *N. mikurensis* in adult and nymphal stages compared to larvae, aligns with previous findings indicating preferential occurrence of *N. mikurensis* infection in mature tick stages [71, 72]. Adult ticks showed higher *Chlamydiales* infection prevalence than nymphs and larvae, consistent with findings from Switzerland, Australia, and Finland [60–62]. In contrast to the above pathogens, *Babesia* spp. infection prevalence showed no significant variation across developmental stages, consistent with observations from Germany and Poland [73, 74]. This pattern may reflect transovarial transmission, in which infected female ticks vertically transmit *Babesia* parasites to their offspring through eggs, resulting in larvae that are already infected at hatching [75, 76].

Assuming that the geographic origins of submitted ticks reflect broader patterns of tick-human encounters recorded through the Tick Prevention app in Switzerland, the spatial analysis of tick-human encounters revealed clustering in populated urban centres and lower elevation areas, predominantly within the Swiss Plateau region. In line with prior studies, this reflects the intersection of human population density, recreational activities, and suitable tick habitat [32, 77, 78]. The positive correlation between sampling effort and TBP detection may suggest that TBP presence broadly correlates with tick abundance at the landscape scale, though we acknowledge that infection prevalence in ticks can vary across localities due to differences in host community composition, microhabitat conditions, and reservoir host density [79–82]. Host community structure plays a critical role in determining both tick density and pathogen prevalence, as only a few widespread vertebrate species (particularly rodents, thrushes, and deer) dominate the maintenance of *I. ricinus* populations and Borrelia transmission cycles, with the relative contribution of different host species varying based on their abundance, body mass, and reservoir competence [83, 84]. Moreover, micro-geographic variation in vegetation characteristics and host habitat use can drive fine-scale differences in both tick density and infection prevalence within forest stands [85]. Nevertheless, the widespread detection of *Borrelia* spp., *Rickettsia* spp., *A. phagocytophilum*, and *N. mikurensis* across diverse geographic regions indicates that the risk of encountering ticks infected with these TBPs is present throughout Switzerland rather than confined to specific high-prevalence areas [19, 86]. This likely results from the mobility of the tick hosts, particularly migratory birds and medium-sized mammals such as deer and rodents, which can transport infected ticks across considerable distances [84, 87–90]. Additionally, the fragmented landscape of Switzerland, with its extensive network of green corridors connecting urban and rural areas, may promote continuous host movement and, in turn, tick dispersal [91–93]. The absence of distinct areas of endemicity contrasts with patterns observed in larger geographic regions such as North America, where Lyme disease risk exhibits clear endemic zones [94, 95], or across continental Europe, where tick-borne encephalitis shows marked spatial heterogeneity with well-defined high-risk areas [96, 97]. This difference may reflect Switzerland's relatively compact geography, in which ecological connectivity facilitates the regular flow of pathogens between tick populations over relatively short distances. This suggests that TBD prevention measures should be promoted uniformly across suitable tick habitats nationwide. The elevational distribution of tick-human encounters is consistent with known ecological constraints on *Ixodes* tick survival and activity [98–100], with encounters being concentrated below 1000 m.

The elevated cumulative TBP burden predicted by our GWR models (30–45% in parts of the Swiss Plateau) exceeds the observed overall prevalence (33.3%), reflecting the aggregation of multiple co-circulating pathogens and spatial clustering in localised hotspots. Previous studies have documented substantial geographic heterogeneity in TBP prevalence across European landscapes, with variation driven by differences in host community composition, microhabitat characteristics, and local environmental conditions [84, 85]. Within Switzerland specifically, earlier surveillance efforts have revealed marked regional variation in infection prevalence for *Borrelia* spp., *Rickettsia* spp., and *N. mikurensis* [19, 54, 55, 101]. Our model-based predictions indicate that specific regions may experience substantially higher TBP exposure risk, though they should be interpreted cautiously given limitations in sampling density and spatial coverage. The GWR models showed variable predictive performance across TBPs. For *Borrelia* spp., predicted infection prevalence ranges generally align with previous findings by Oechslin et al. [19] and Pilloux et al. [17], who reported 9.7–18.5% in western regions and 14.5% in Zurich. However, their observed infection prevalence in Bern (35.8%) substantially exceeded our predicted range, whilst Basel area findings (7.2–17.1%) showed partial correspondence with our predictions. Our *Borrelia* spp. infection prevalence predictions align with those of Casati et al. [102], who reported 15% in Ticino and 22.5% in western Switzerland, suggesting consistency across both citizen science and traditional surveillance approaches in these regions. Similarly, Oechslin et al. [19] found the highest *B. miyamotoi* infection prevalence in western regions, consistent with our model. *Rickettsia* spp. predictions showed a north-south gradient, matching Oechslin et al.'s [19] findings of the highest infection prevalence in Basel and Zurich. This convergence among *Rickettsia* spp. may suggest that spatial infection patterns are driven by underlying ecological factors, such as climate, vegetation, or reservoir-host distributions, rather than sampling methodology. Our western Switzerland predictions also correspond with Lommano et al.'s [55] observations of 10.2%. For *N. mikurensis*, spatial predictions align with Maurer et al.'s [54] reported rates of 0.9–4.7% in northeastern Switzerland and Lommano et al.'s [55] observation of 6.4% in western areas.

Peak encounters and TBP detections during May–June align with established temperate climate tick activity patterns and validate prior encounter data from Switzerland [19, 32, 103, 104]. Analysis of temporal infection prevalence patterns for individual TBPs in submitted ticks revealed that whilst the majority exhibited peak infection prevalence during the late spring and summer months, *A. phagocytophilum* demonstrated a distinctive seasonal pattern; specifically, its infection prevalence reduced during the summer months across all 3 study years, characterised by bimodal peaks during late spring and early autumn. This bimodal pattern, with peaks in late spring and early autumn, may reflect the seasonal questing activity of *I. ricinus*, which exhibits two annual activity peaks corresponding to periods of optimal temperature and humidity [98–100]. The observed *A. phagocytophilum* infection pattern may result from seasonal variation in tick developmental stage activity, shifts in reservoir host (primarily ungulates) availability and habitat use [83–85], and temperature-dependent effects on pathogen transmission efficiency [105–107]. This contrasts with the predominantly spring–summer pattern reported for human anaplasmosis cases in the USA [108]. Given the limited number of *A. phagocytophilum*-positive samples in our study (*n* = 19), these observations require cautious interpretation.

Several limitations should be acknowledged when interpreting our findings. The citizen science approach, whilst enabling large-scale data collection, may introduce a sampling bias, as participation was voluntary and geographically clustered around urban centres. However, our findings align with those from active sampling studies [19, 103, 104], suggesting that potential sampling biases may not substantially affect their validity. Moreover, geographic representation was similarly uneven, with very few specimens reported from above 1000 m elevation, limiting our statistical power to detect elevational gradients in TBP infection prevalence. The uneven temporal distribution of submissions, particularly the four-fold reduction in the number of specimens collected in 2019 compared to 2018 and 2020, limits the ability to resolve temporal patterns accurately. Additionally, the developmental stage composition was heavily skewed towards nymphs, which could have influenced stage-specific infection prevalence data. The poor predictive performance observed for low-prevalence TBPs such as *Babesia* spp., *A. phagocytophilum*, *Chlamydiales*, and *F. tularensis* is probably linked to insufficient positive cases for robust spatial modelling. Finally, the inconsistent testing across study years, with some TBPs examined only in specific years, including *C. burnetii* in 2019–2020 and *R. helvetica* and *Rhabdochlamydia* in 2018, has affected temporal comparisons and overall infection prevalence estimates.

Despite the inherent sampling biases associated with voluntary participation, our citizen science approach demonstrated consistency with previous active surveillance studies [19, 103, 104] across Switzerland, validating its utility for large-scale TBP monitoring in similar settings. Future research to build upon this foundation may benefit from integrating complementary sampling strategies that combine the broad geographical coverage achieved through citizen science with targeted systematic sampling in under-represented ecological zones, particularly montane environments above 1000 m elevation. The Tick Prevention app framework could be enhanced to capture additional contextual data to enable more complex epidemiological analyses, including specific land-cover types where tick encounters occur and recreational activities associated with tick exposure. This enhanced dataset would facilitate the investigation of risk factors and exposure patterns across different landscape types and environmental contexts. Expanding the citizen science model to include detection and monitoring of emerging tick species, such as *Hyalomma* spp., would provide early warning capabilities for novel TBD threats as climate change facilitates range expansions.

## Conclusions

This study highlights the public health relevance of TBP surveillance in Switzerland, where one-third of examined ticks harboured at least one TBP. Spatial models predicted that cumulative TBP infection prevalence in submitted ticks could reach 30–45% in focal areas of the Swiss Plateau, consistent with documented spatial heterogeneity in TBP distribution across European forests. These represent region-specific estimates that should be interpreted alongside the observed national average of 33.3%. Citizen science approaches for tick and TBP surveillance may be highly valuable for large-scale monitoring programmes, successfully generating data consistent with traditional surveillance methods whilst engaging the public in disease prevention efforts. Similar citizen science initiatives in other countries, including the US and Finland's nationwide research station network, have previously demonstrated success in capturing spatiotemporal patterns of tick-human encounters and associated pathogen prevalence across large geographic areas [109, 110]. Such platforms demonstrate the capacity to capture human tick exposure patterns across diverse geographical regions, complementing traditional field sampling with human-centred exposure data. The integration of digital platforms with epidemiological surveillance represents a scalable and cost-effective approach that could be adapted to other vector-borne disease monitoring systems. This is especially important as climate change continues to influence tick distribution and activity patterns. The exhibited effectiveness of citizen science in TBP surveillance provides a scalable model that could be adopted by other countries facing similar TBD challenges, supporting broader European surveillance networks and contributing to continental-scale understanding of TBP epidemiology. Finally, the identification of significant co-infection patterns, particularly the association between *Borrelia* spp. and *N. mikurensis*, suggests complex ecological interactions within TBP communities that warrant further investigation.

## Supplementary Information

Supplementary Material 1.

Supplementary Material 2.

Supplementary Material 3.

Supplementary Material 4.

Supplementary Material 5.

Supplementary Material 6.

Supplementary Material 7.

## Data Availability

The raw data, including geographical coordinates and encounter dates collected through the “Tick Prevention” app, will be made freely available upon publication of this manuscript. The dataset can be accessed via the research group’s webpage at the following link: https://www.chuv.ch/en/microbiologie/imu-home/research/research-groups/gilbert-greub/supplementary-data
