# Supplementary material for: Surveillance of tick-borne pathogens in ticks collected from Swiss residents
Source: Parasit Vectors. 2026 May 4;19:255. doi: 10.1186/s13071-026-07363-8 (PMC13285383; doi:10.1186/s13071-026-07363-8)
Supplement: Supplementary file 1 — Supplementary Material 1. [file 13071_2026_7363_MOESM1_ESM.docx]

**Surveillance of Tick-Borne Pathogens in Ticks Collected from Swiss Residents**

Running title: Tick-Borne Pathogens in Swiss Ticks

Ehsan Ghasemian^1*^, Bastian Marquis^1^, Florian Tagini^1^, Sébastien Aeby^1^, Werner Tischhauser^2^, Reto Lienhard^3,4^, Christian Beuret^5^, Virginie Martin^1^, Silvan Hälg^6,7^, Pie Müller^6,7^, Antony Croxatto^3,4^, Onya Opota^1^, Gilbert Greub^1,4 *^

^1^ Institute of Microbiology, University Hospital Center and University of Lausanne, Lausanne, Switzerland

^2^ ZHAW Spin-off “A&K Strategy Ltd”, Smartphone-App “Zecke – Tick Prevention” Neuhaus SG, Switzerland

^3^ ADMED microbiologie, Boucle de Cydalise 16, 2300 La Chaux-de-Fonds, Switzerland

^4^ National Reference Center for Tick-transmitted Diseases, Lausanne and La Chaux-de-Fonds, Switzerland

^5^ Spiez Laboratory, Federal Office for Civil Protection, Spiez, Switzerland

^6^ Swiss Tropical and Public Health Institute, Allschwil, Switzerland

^7^ University of Basel, Basel, Switzerland

*Correspondence:

Ehsan Ghasemian, Institute of Microbiology, University of Lausanne and University Hospital Center, Rue du Bugnon 48, CH-1011 Lausanne, Switzerland

[ehsan.ghasemian@chuv.ch](mailto:ehsan.ghasemian@chuv.ch), Tel: +41 (0)21 314 40 97

Professor Gilbert Greub, Institute of Microbiology, University of Lausanne and University Hospital Center, Rue du Bugnon 48, CH-1011 Lausanne, Switzerland

[gilbert.greub@chuv.ch](mailto:gilbert.greub@chuv.ch), Tel: +41 (0)21 314 49 79

Keywords: Tick, Tick-borne disease, Prevalence, Spatial epidemiology, Switzerland, *Borrelia* spp., *Rickettsia* spp., *Chlamydiales*

Category: Original Article

**Supplementary Methods**

*TBP infection prevalence*

TBP prevalence was calculated and statistically analysed using R (v4.4.2) and Rstudio (v2024.09.1) with the ggplot2 (v3.5.2), RcolorBrewer (v1.1.3), scales (v1.4.0), and openxlsx (v4.2.8) packages [1–6]. For each TBP, infection prevalence in submitted ticks was computed as the proportion of positive samples relative to the total sample size, with 95% confidence intervals estimated using exact binomial methods (binom.test()).

Co-infection patterns amongst TBPs were analysed using dplyr (v1.1.4), openxlsx, and ComplexHeatmap (v2.22.0) packages [7–9]. Pairwise TBP associations were evaluated using Fisher's exact tests applied to 2X2 contingency tables. Results were visualised using a triangular heat map, with colour intensity indicating statistical significance.

*TBP infection prevalence across tick developmental stages*

TBP infection prevalence across tick developmental stages was analysed using dplyr, tidyr (v1.3.1), and ggplot2 packages [10]. Pairwise comparisons between all developmental stages were conducted using Fisher's exact tests (α=0.05). Infection prevalence data were visualised using stacked bar charts showing the proportion of infected relative to uninfected ticks within each developmental stage.

*Geospatial and regional analysis of tick-human encounters*

Geographic coordinates from tick collection locations were spatially matched to Swiss postal code regions using the sf (v1.0.21), readr (v2.1.5), dplyr, ggplot2, ggspatial (v1.1.9), and viridis (v0.6.5) packages [11–15]. Latitude and longitude coordinates were validated and filtered to ensure values fell within Swiss territorial boundaries (45.8°-47.9°N, 5.9°-10.6°E). Coordinate data were converted to spatial features using st_as_sf() with WGS84 projection (CRS 4326) and subsequently transformed to the Swiss national coordinate reference system LV95 (EPSG:2056) using st_transform() to match postal code shapefile projections. Spatial intersection analysis was performed via st_join() to assign 4-digit postal codes to each sampling location based on point-in-polygon relationships. Population data for two-digit postal regions were derived from the Swiss Federal Statistical Office (BFS) postal code-level population statistics (Swiss Federal Statistical Office, 2024), where four-digit postal codes were aggregated by their two-digit prefix to calculate total counts for each regional area.

To examine the spatial distribution of tick encounters across Swiss postal regions, data on received ticks were aggregated by two-digit postal code prefixes and normalised per 10,000 inhabitants. Geographic visualisation of tick distribution across Swiss postal regions was performed using packages sf, ggplot2, and ggspatial. Population density was calculated as inhabitants per 10 km^2^ for each two-digit postal region using area calculations from the spatial geometries (st_area() function) and official BFS population data. A continuous gradient map was created displaying population density using a square-root transformed colour scale. Tick sample locations were overlaid at postal region centroids, with marker size proportionally scaled to represent tick count magnitude.

Spatial density patterns of tick samples were subsequently visualised using two-dimensional kernel density estimation, overlaid on topographic elevation data. Final cartographic outputs, with descriptive statistics including frequency distributions and spatial coverage summaries computed to assess geographic representation and identify clustering patterns across the dataset. Additionally, elevation values for each sample location were extracted from digital elevation model raster data, with samples categorised into 200-metre elevation bins using floor division (floor(Elevation_m / 200) * 200) to standardise altitudinal groupings. Sample and TBP infection prevalence in submitted ticks was calculated for each altitudinal stratum. Pearson correlation coefficients between individual TBP infection prevalence and elevation were computed using cor() with pairwise complete observations to handle missing data. Linear regression models (lm()) were fitted for each TBP infection prevalence against elevation to assess statistical significance of altitudinal trends.

*Geospatial and regional analysis of TBP infection prevalence*

The relationship between number of ticks and TBP detection rates was assessed at the two-digit postal region level. Pearson product-moment correlation analysis was performed using cor.test() with complete observations to quantify the linear relationship between regional sample size and total TBP counts. The correlation coefficient, *P*-value, and significance level were calculated and reported, with statistical significance determined at α=0.05. A scatter plot was constructed using ggplot2 package using geom_smooth() with method "lm".

Spatial analysis of TBP infection prevalence across Switzerland was conducted using a grid-based Geographically Weighted Regression (GWR) approach. Only TBPs with ≥10 positive samples were included in subsequent analyses for model reliability. Individual tick sampling locations with geographic coordinates (latitude, longitude) were aggregated into regular square grid cells superimposed over Switzerland. Sample points were assigned to grid cells using spatial intersection operations, and TBP infection prevalence was calculated for each grid cell as the proportion of positive samples amongst all tested samples within that cell. This aggregation was performed using the sf package for spatial data handling and dplyr for data manipulation. For each TBP individually, optimal grid cell sizes were determined by testing multiple grid resolutions (2, 5, 7, 10, 15, 20, 25, and 30 km). Grid size selection was based on a composite accuracy score incorporating four statistical criteria: (*i*) Akaike Information Criterion (AIC) for model fit, (*ii*) global R^2^ for explanatory power, (*iii*) cross-validation mean squared error for predictive accuracy, and (*iv*) spatial autocorrelation of residuals approximated using Moran's I statistic. The accuracy score was calculated as a weighted combination assigning 30% weight to AIC (model parsimony), 30% to R² (explanatory power), 20% to cross-validation error (predictive accuracy), and 20% to spatial autocorrelation penalty (assumption validity), with weights chosen to prioritise model fit and explanatory power whilst maintaining adequate predictive performance and spatial independence of residuals. Normalisation was applied relative to each TBP's specific value ranges to ensure comparable scoring across TBPs. Spatial regression modelling was performed using GWR with the spgwr package (v0.6.37) [16], treating TBP infection prevalence as the dependent variable and geographic coordinates as independent variables. For each TBP, GWR was applied using the individually determined optimal grid size. Adaptive kernel bandwidth was selected using AIC optimisation (bw.gwr function), and GWR models were fitted using Gaussian kernels (gwr.basic function). Visualisation was implemented using ggplot2 package. Switzerland country boundaries were obtained using the rnaturalearth package (v1.0.1) [17].

Geographic variation in TBP infection prevalence across Swiss postal regions was evaluated. For individual TBPs, associations between TBP presence and two-digit postal regions were assessed using chi-square tests of independence or Fisher's exact tests (10,000 Monte Carlo simulations) when expected cell frequencies were below five. Post-hoc analyses employed Fisher's exact tests comparing each region against all other regions combined, with both two-sided and one-sided alternatives to identify regions with significantly elevated infection prevalence. For total TBP counts, regional differences were evaluated using Kruskal-Wallis tests as the primary non-parametric approach, which is appropriate for non-normally distributed count data. Pairwise post-hoc comparisons employed Wilcoxon rank-sum tests (Mann-Whitney U) comparing individual regions against pooled data from all other regions. As an alternative approach, Poisson regression models were fitted to account for the discrete count nature of pathogen data whilst testing for regional effects.

*Temporal trends in TBP detection and tick-human encounters*

Temporal patterns of TBP detection were analysed using dplyr, ggplot2, lubridate (v1.9.4) [18], tidyr, and scales packages. Visualisation approaches included stacked area plots displaying cumulative TBP burden over time and faceted plots showing individual TBP temporal trends with custom colour palettes for consistent identification.

Temporal patterns of tick-human encounter and TBP infection prevalence were analysed using the dplyr, openxlsx, and purrr (v1.0.4) [19] packages. Monthly TBP infection prevalence rates were calculated with 95% confidence intervals using Wilson score intervals. Statistical significance of temporal patterns was assessed using multiple approaches: chi-square tests for overall monthly distributions, and Fisher's exact tests (with Monte Carlo simulation, 10,000 iterations when necessary) when any expected frequency was less than five. Post-hoc analyses included Dunn tests for significant results and pairwise month comparisons using two-proportion *z*-tests with Bonferroni correction for multiple comparisons. Temporal infection prevalence differences were evaluated using chi-square tests or Fisher's exact tests when cell counts were insufficient. Monthly tick collection data were analysed using chi-square goodness of fit tests to assess whether tick abundance was equally distributed across months throughout the year. Individual month *P*-values were computed using two-tailed *z*-tests based on standardised residuals.

Monthly infection rates were analysed using two complementary approaches. First, overall monthly infection prevalence was tested for temporal variation using chi-square tests or Fisher's exact tests when expected cell frequencies were below five. Second, to account for the influence of monthly tick abundance on infection patterns, a standardised residual analysis was performed comparing observed monthly infection counts against expected counts based on overall infection rates and monthly tick collection numbers. This approach isolated months with infection rates significantly higher or lower than expected based on tick-human encounter alone. Standard errors were calculated using binomial distribution assumptions, and individual month *P*-values were computed using normal approximation to the binomial distribution. For TBPs with sufficient positive cases (≥10), chi-square tests or Fisher's exact tests (with Monte Carlo simulation, 10,000 iterations when necessary) were applied to test for non-uniform temporal distribution. TBPs showing significant overall temporal patterns (*P* < 0.05) were subjected to month-specific analysis using standardised residuals, with individual month *P*-values calculated to identify peak transmission periods. For significant overall patterns, post-hoc pairwise comparisons between months were conducted using proportion tests with Bonferroni correction for multiple comparisons.

Temporal relationships between tick-human encounter and TBP detection rates were evaluated at the month-year level using the dplyr, ggplot2, and gridExtra (v2.3) [20] packages. Pearson product-moment correlations were done to quantify linear relationships between the intensity of tick-human encounters and TBP detection frequencies.

**Supplementary Results**


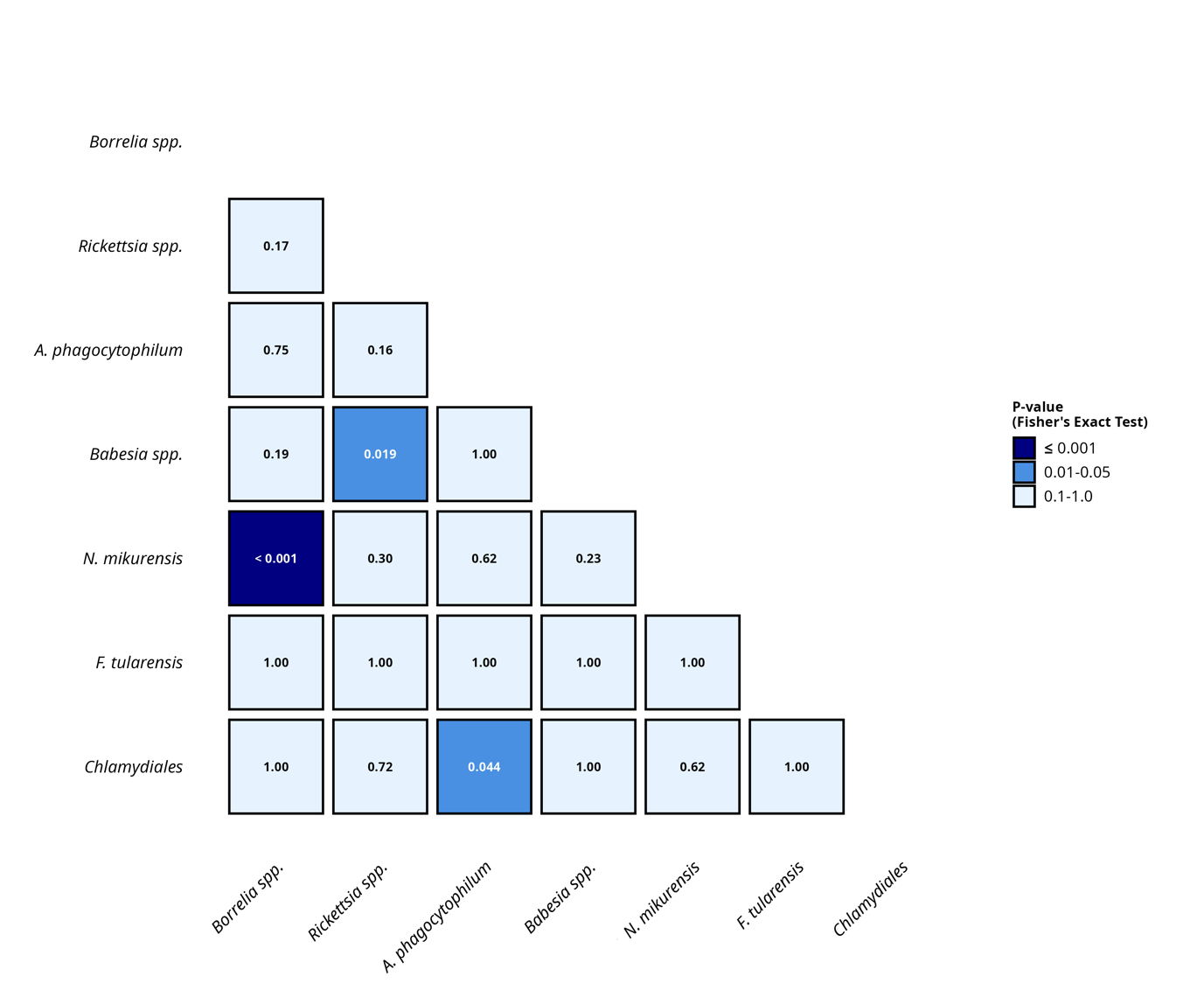


Figure S1. Pairwise statistical associations between TBPs based on co-occurrence patterns. The triangular heatmap displays *P*-values from Fisher's exact tests conducted on 2×2 contingency tables for TBP pairs. *P*-values are displayed within each cell, with white text indicating statistically significant associations (*P* < 0.05) and black text for non-significant relationships.

*C*. *burnetii* and *R. helvetica* are excluded from this analysis due to temporal limitations in screening protocols (see Methods section for details). *Borrelia miyamotoi* and *B. microti* are excluded to prevent misinterpretation, as their respective genus-level taxa (*Borrelia* spp. and *Babesia* spp.) are included in the analysis.


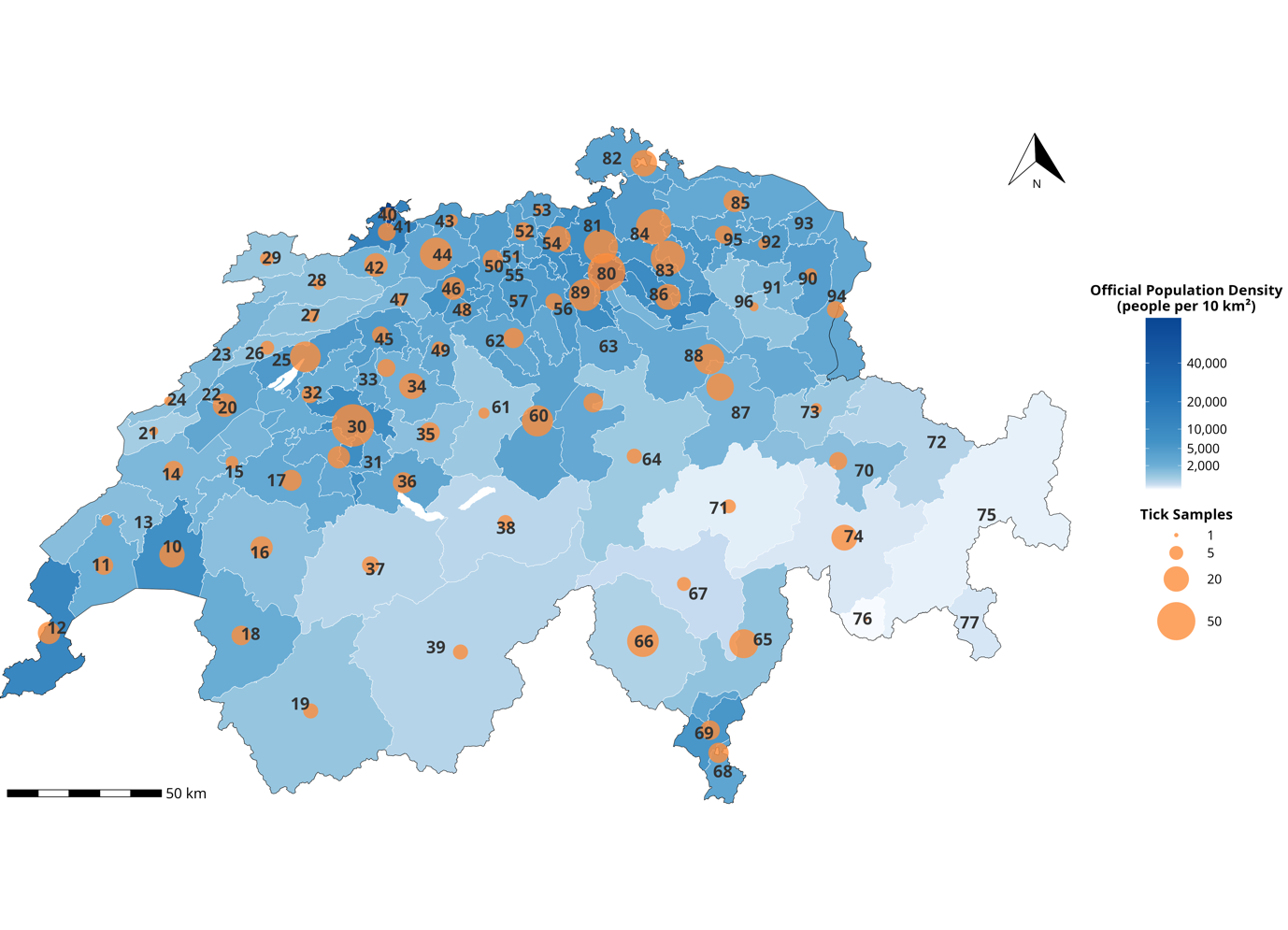
**Figure S2.** Spatial distribution of tick encounters across Swiss postal regions with population density context. Map displays official population density per 10 km^2^ by two-digit postal code regions (blue gradient scale, square-root transformed) overlaid with tick sample counts (orange circles, size proportional to sample number). Population density data derived from Swiss Federal Statistical Office (BFS) postal code-level statistics. Two-digit postal codes are labelled within each region. Scale bar and north arrow provided for reference. White boundaries delineate postal regions, with black outline showing Switzerland's national border.

**
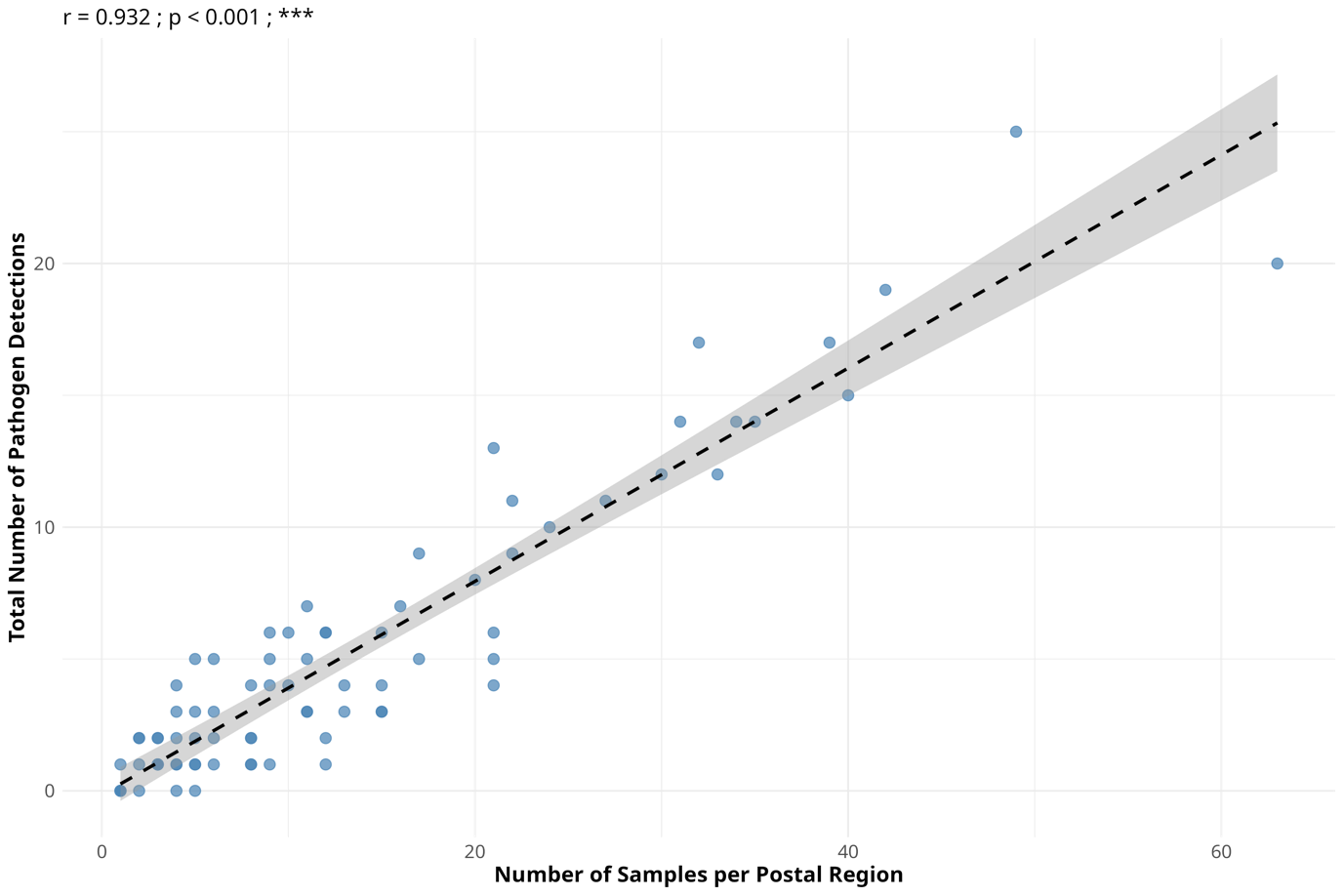
Figure S3. Relationship between number of ticks and aggregated TBP across Swiss postal regions.** Each point represents a two-digit postal code region, with the x-axis showing the total number of tick samples collected within that region and the y-axis displaying the cumulative number of TBP detections. The black dashed line represents the linear regression fit with 95% confidence interval (grey shading). Correlation statistics are displayed in the subtitle, including Pearson's correlation coefficient (r), *P*-value, and significance level (*** *P* < 0.001).

*C*. *burnetii* and *R. helvetica* are excluded from this analysis due to temporal limitations in screening protocols (see Methods section for details). *B. miyamotoi* and *B. microti* are excluded to prevent misinterpretation, as their respective genus-level taxa (*Borrelia* spp. and *Babesia* spp.) are included in the analysis.


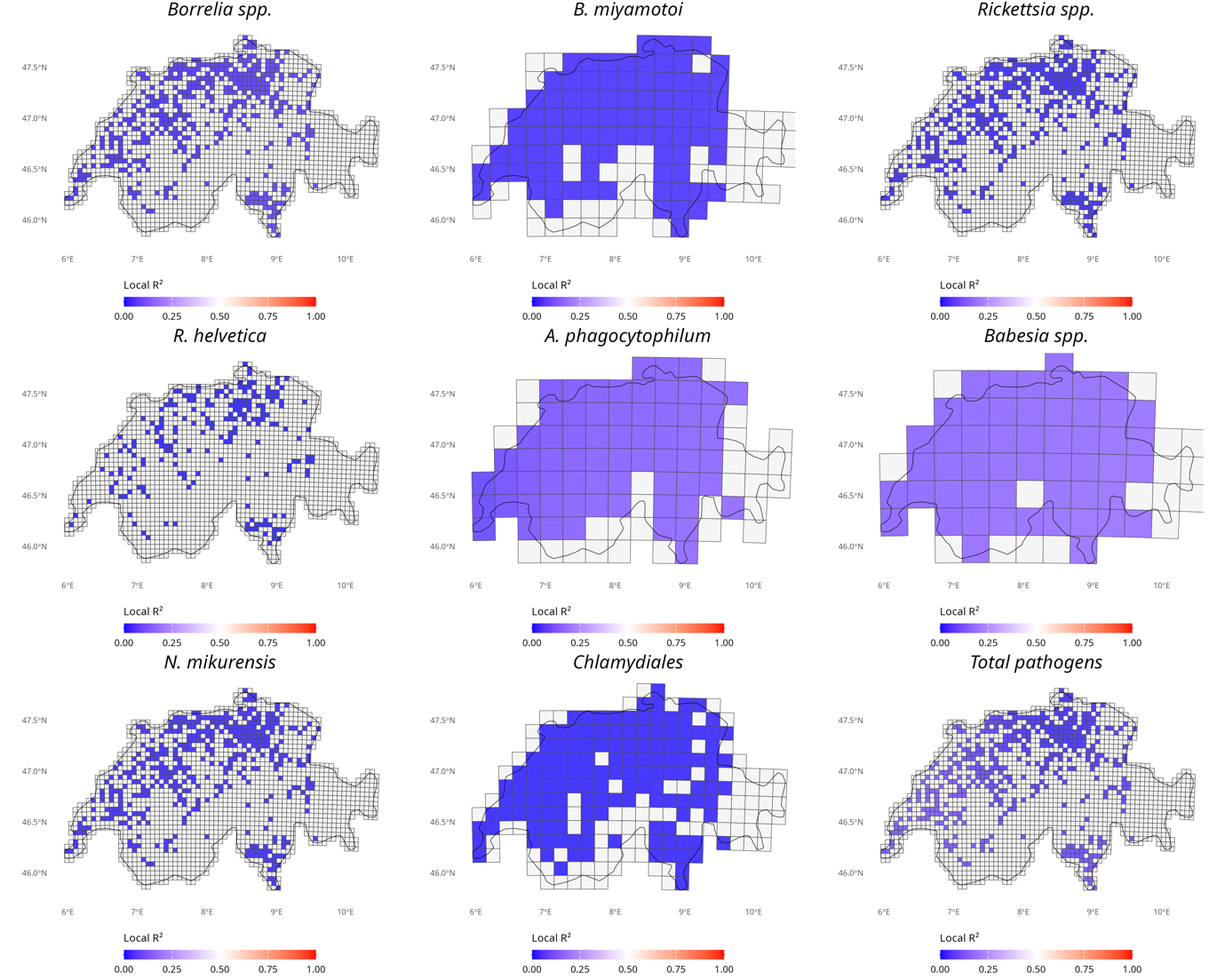


**Figure S4: Local Model Performance (Local R^2^) from GWR analysis.** Spatial variation in local model performance across Switzerland, showing local R^2^ values from GWR models for each TBP. Local R^2^ represents the proportion of prevalence variance explained by the GWR model within the local neighborhood of each grid cell, indicating how well the geographic coordinate-based model captures spatial patterns at different locations. Values range from 0 (blue, poor local model fit) to 1 (red, excellent local model fit), with white representing moderate fit (R^2^ ≈ 0.5). Grid Resolution: Each pathogen uses its individually optimised grid cell size (ranging from 5-30 km) determined through grid size optimisation.

*R. helvetica* predictions are based on 2018 specimens due to temporal screening limitations (see Methods section for details). Only TBPs with ≥10 positive samples across the entire dataset were included to ensure sufficient statistical power for reliable spatial modeling.

**
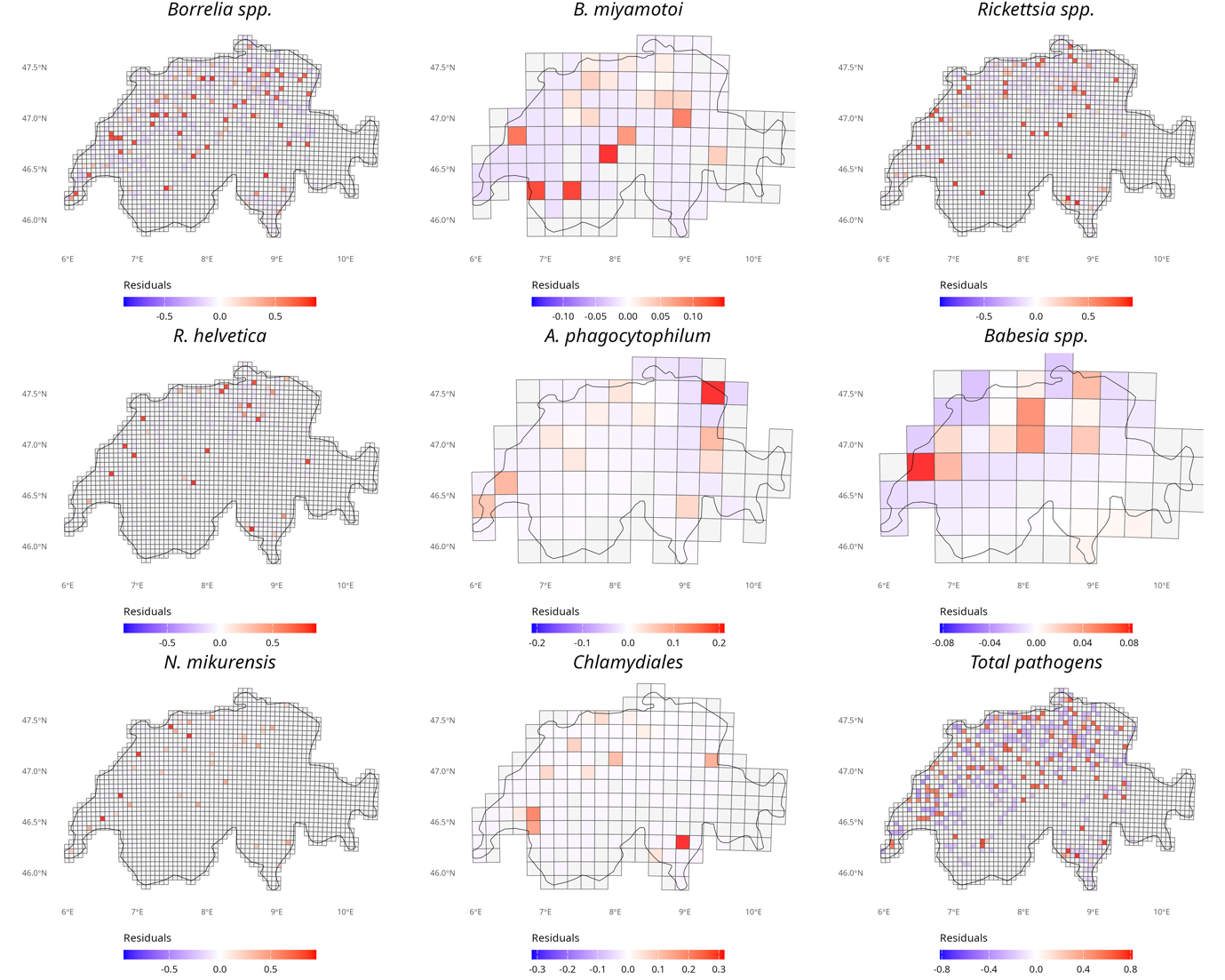
**

**Figure S5: Model residuals from GWR analysis.** Spatial distribution of model residuals (prediction errors) from GWR analysis, calculated as observed prevalence minus predicted prevalence for each grid cell. Residuals are displayed using a diverging colour scale where blue indicates negative residuals (model over-prediction), white indicates residuals near zero (accurate predictions), and red indicates positive residuals (model under-prediction). Grid Resolution: Each pathogen uses its individually optimised grid cell size (ranging from 5-30 km) determined through grid size optimisation.

*R. helvetica* predictions are based on 2018 specimens due to temporal screening limitations (see Methods section for details). Only TBPs with ≥10 positive samples across the entire dataset were included to ensure sufficient statistical power for reliable spatial modeling.

**
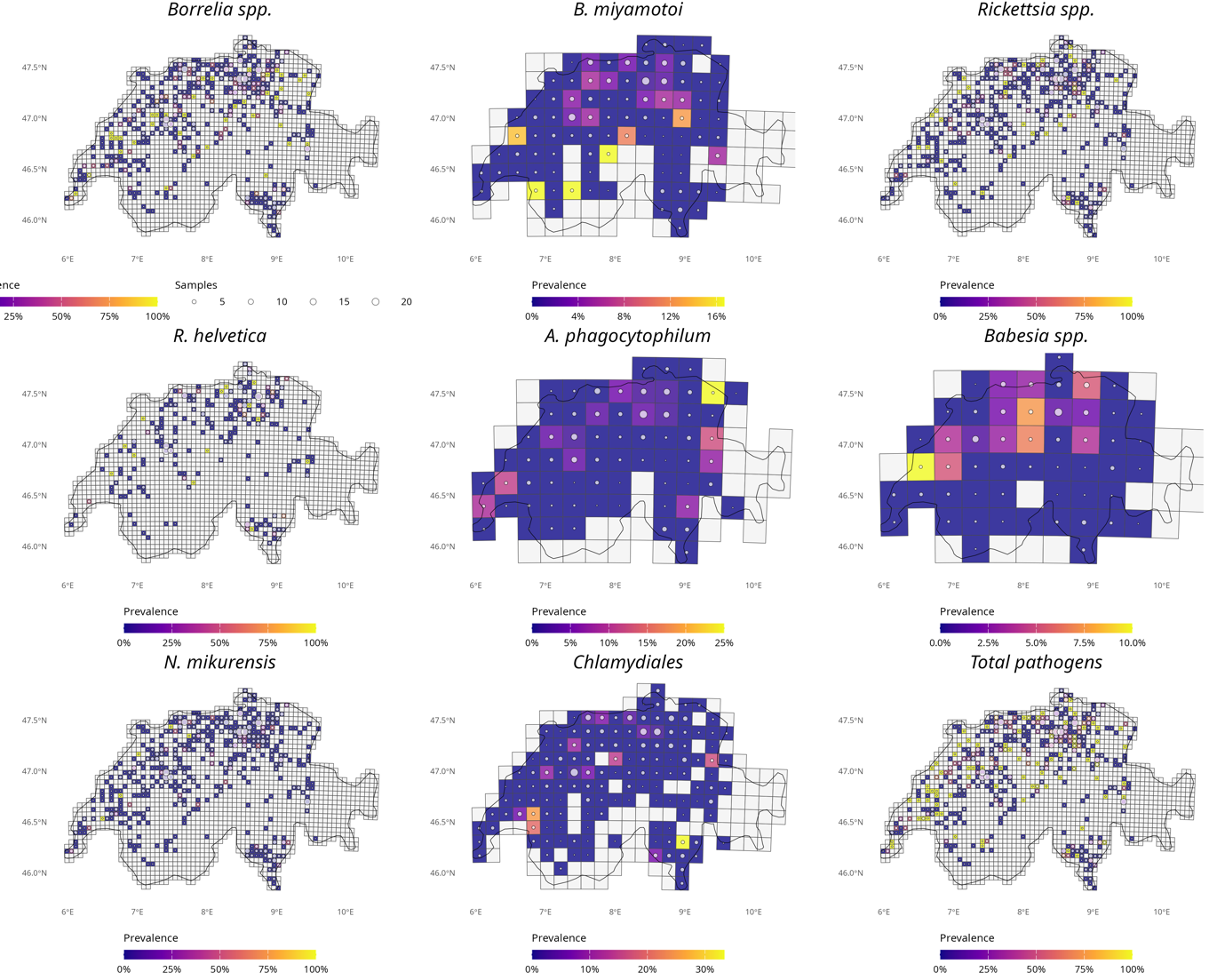
**

**Figure S6. Observed prevalence of TBPs across Switzerland.** Spatial distribution of observed prevalence for TBPs across Switzerland using TBP-specific optimal grid sizes determined through grid size optimisation. Each subplot represents a different pathogen. White circles overlaid on the maps represent sampling locations, with circle size proportional to the total number of tick samples tested in each grid cell. Grid Resolution: Each pathogen uses its individually optimised grid cell size (ranging from 5-30 km) determined through grid size optimisation. Missing Data: Gray areas indicate grid cells with insufficient data for analysis (fewer than required minimum samples).

*R. helvetica* predictions are based on 2018 specimens due to temporal screening limitations (see Methods section for details). Only TBPs with ≥10 positive samples across the entire dataset were included to ensure sufficient statistical power for reliable spatial modeling.

**
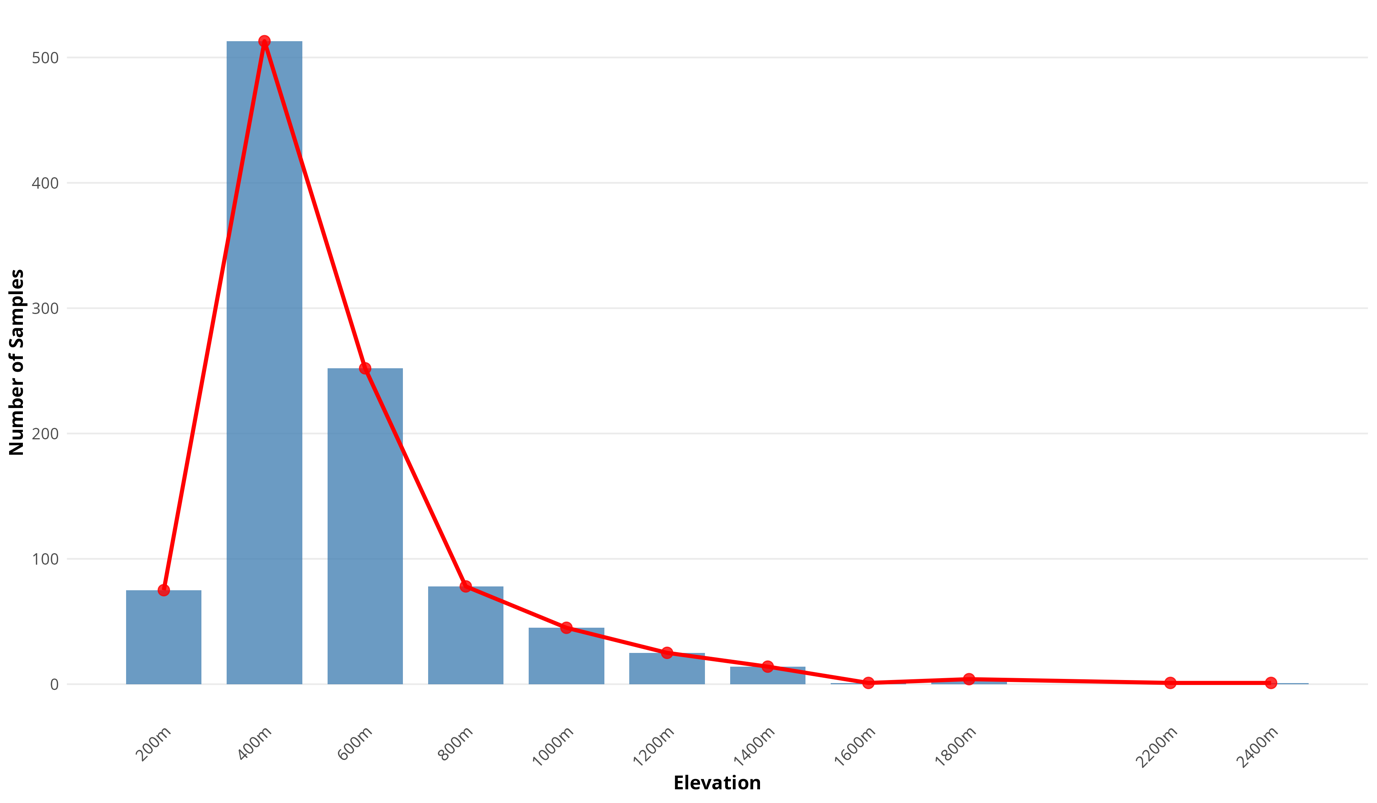
Figure S7. Distribution of tick samples across elevation gradients in Switzerland.** Blue bars represent the number of samples received within each 200-metre elevation bin, whilst the red line and points illustrate the overall sampling pattern across the altitudinal gradient.

**
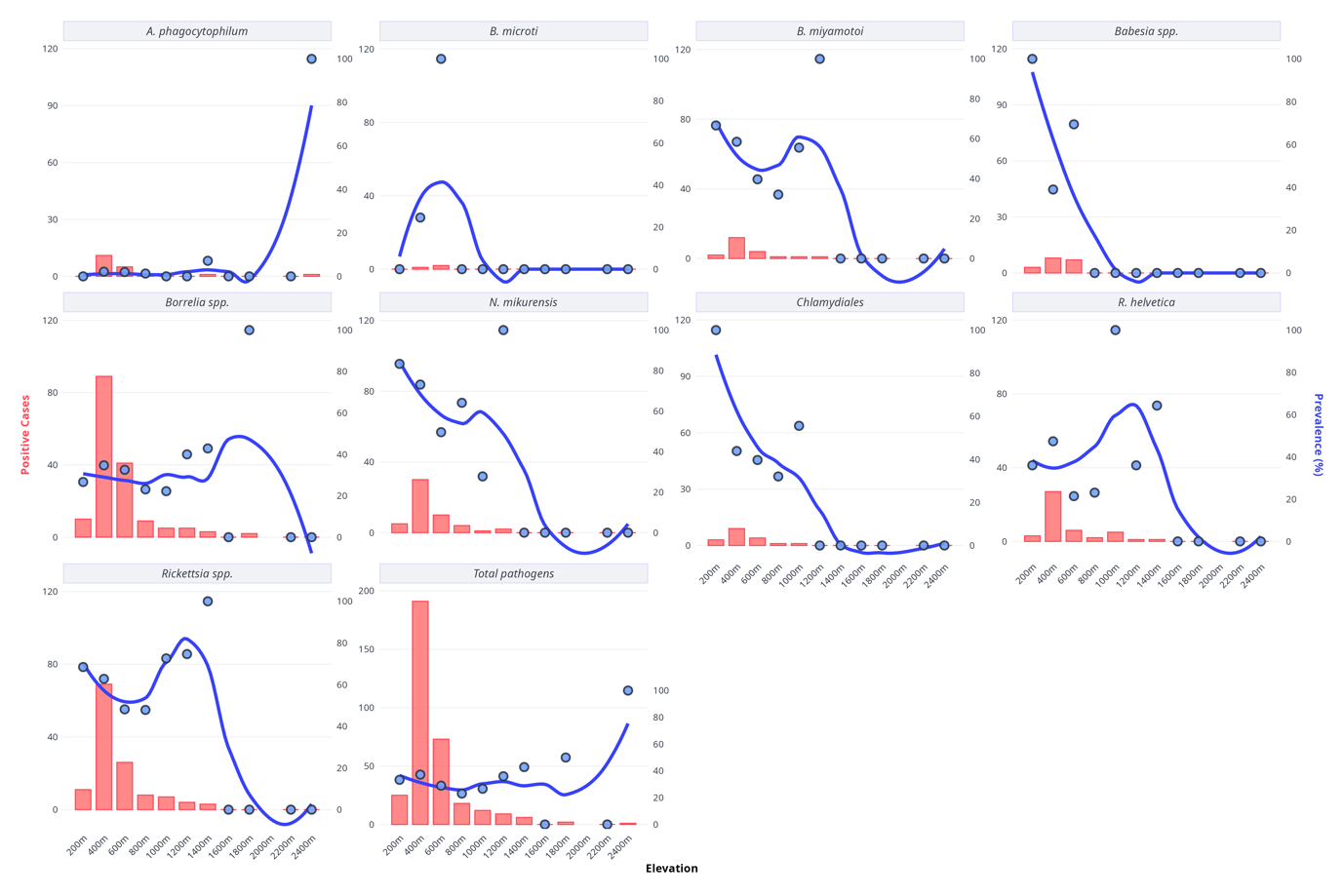
**

**Figure S8. Distribution of the most prevalent TBPs across elevation gradients.** Multi-panel visualisation displaying (*i*) the top nine most common TBPs ranked by total positive case counts, and (*ii*) aggregating TBP. Red bars indicate the absolute number of positive ticks within each 200-metre elevation bin (left y-axis), whilst blue trend lines with overlaid points show prevalence patterns fitted using LOESS smoothing (right y-axis, scaled). This dual-metric approach enables simultaneous assessment of both the absolute TBP burden and relative prevalence trends across Switzerland's altitudinal gradient, revealing elevation-dependent patterns in TBP distribution


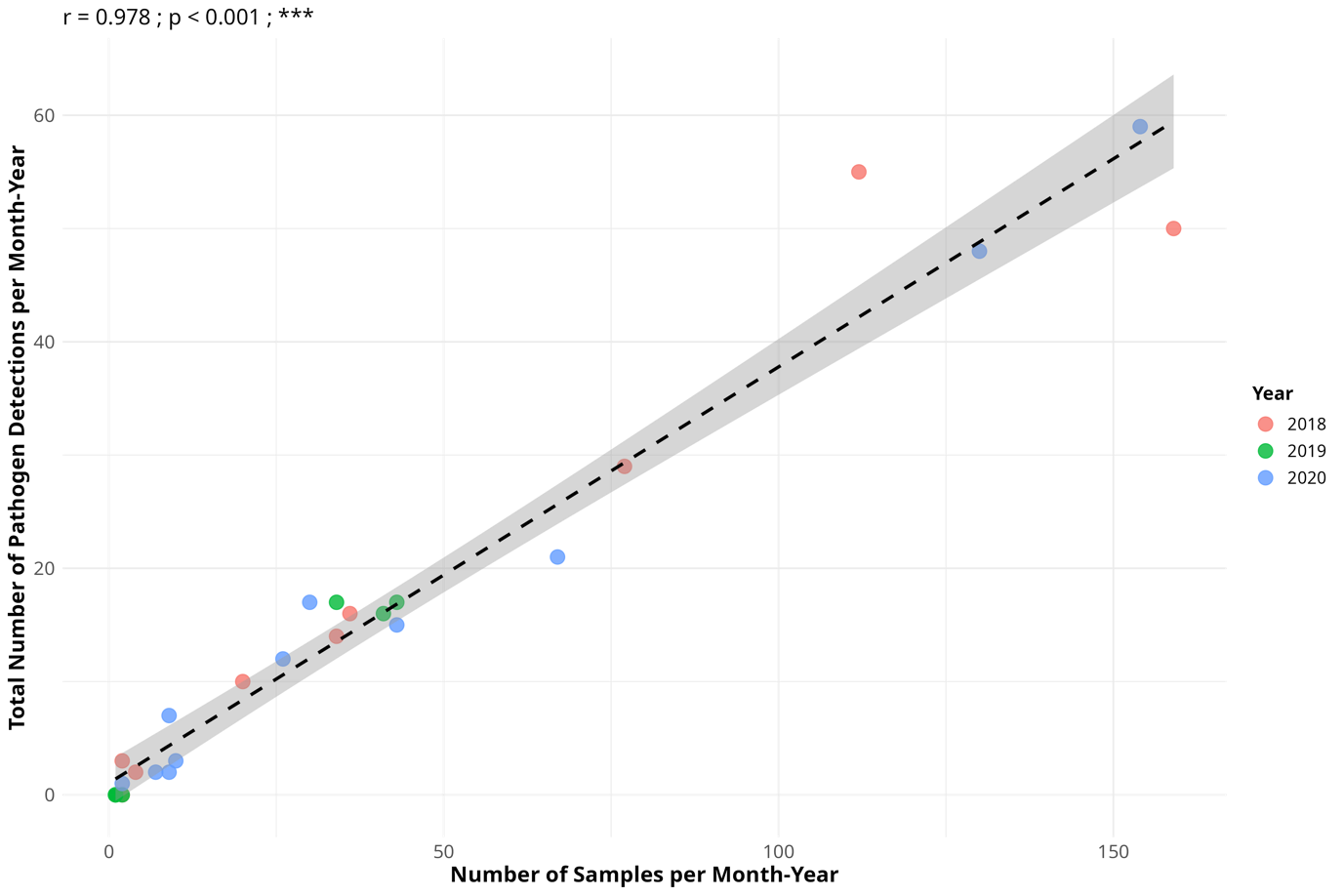


**Figure S9. Relationship between monthly tick-human encounter and aggregated TBP across the study period.** Each point represents a unique month-year combination, with the x-axis showing the total number of tick samples collected and the y-axis displaying the cumulative number of TBP detections. The black dashed line represents the linear regression fit with 95% confidence interval (grey shading). Correlation statistics are displayed in the subtitle, including Pearson's correlation coefficient (r), *P*-value, and significance level (*** *P*<0.001).

*C*. *burnetii* and *R. helvetica* are excluded from this analysis due to temporal limitations in screening protocols (see Methods for details). *B. miyamotoi* and *B. microti* are excluded to prevent misinterpretation, as their respective genus-level taxa (*Borrelia* spp. and *Babesia* spp.) are included in the analysis.


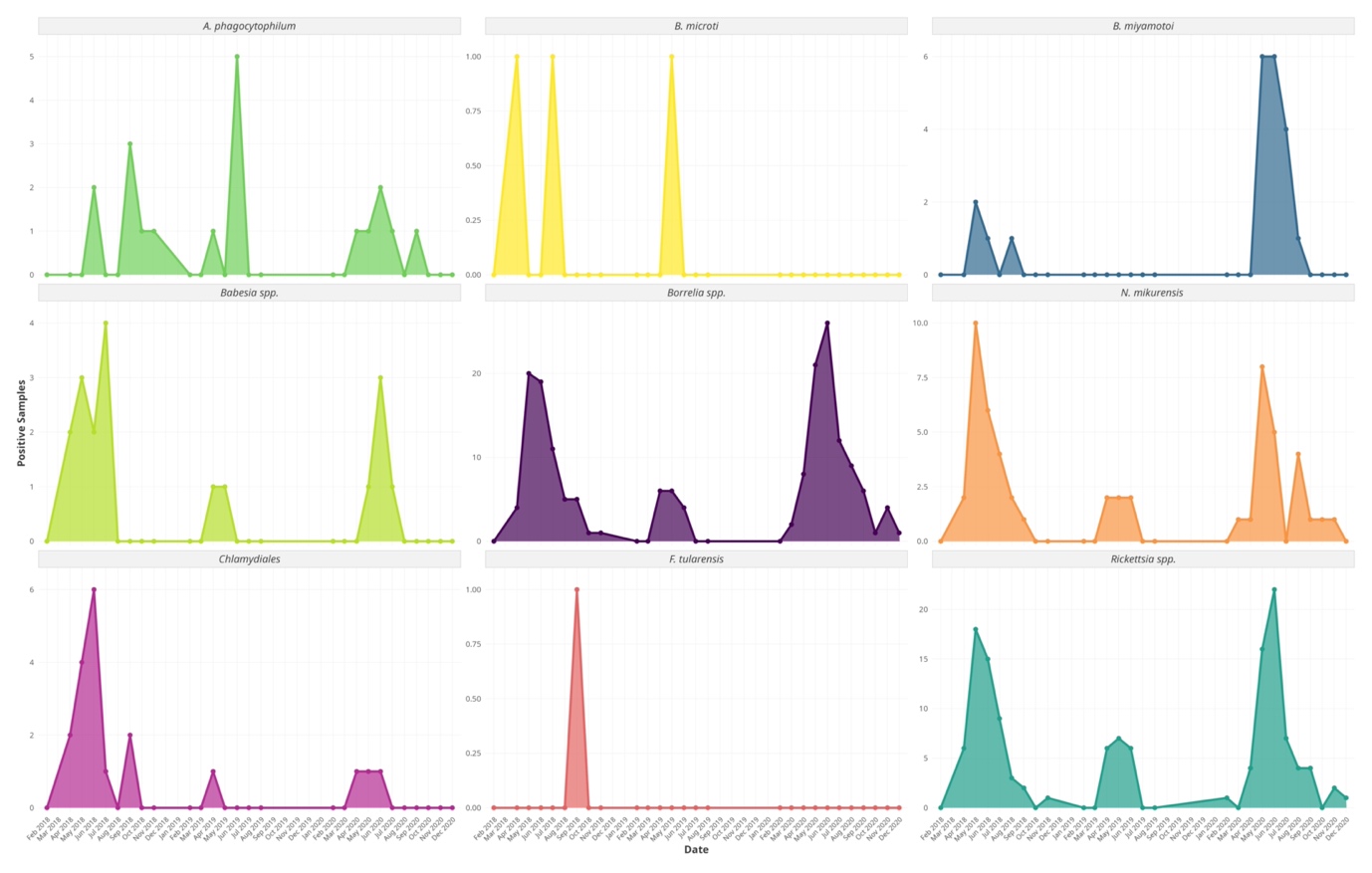


**Figure S10. Temporal distribution of individual TBP detections across the study period.** Each panel displays the monthly detection pattern for a specific TBP. The filled areas represent cumulative positive detections per month, with overlaid lines and points showing the temporal trend and individual monthly data points, respectively. The y-axis scale is independently adjusted for each TBP panel to optimise visualisation of individual temporal patterns, whilst the x-axis maintains consistent monthly intervals across all panels. Each TBP is colour-coded consistently throughout the analysis, enabling direct comparison of seasonal detection patterns and temporal dynamics.

*C. burnetii* and *R. helvetica* are excluded from this analysis due to temporal limitations in screening protocols (see Methods section for details).

**Supplementary References**

1. R Core Team (2024). _R: A Language and Environment for Statistical Computing_. R Foundation for Statistical Computing, Vienna, Austria. <https://www.R-project.org/>.

2. Posit team (2025). RStudio: Integrated Development Environment for R. Posit Software, PBC, Boston, MA. URL http://www.posit.co/.

3. Wickham H (2016). ggplot2: Elegant Graphics for Data Analysis. Springer-Verlag New York. ISBN 978-3-319-24277-4, https://ggplot2.tidyverse.org.

4. Neuwirth, E. (2014) RColorBrewer: ColorBrewer Palettes. R Package Version 1.1-3. https://CRAN.R-project.org/package=RColorBrewer.

5. Wickham H, Pedersen T, Seidel D (2025). scales: Scale Functions for Visualization. R package version 1.4.0, https://scales.r-lib.org.

6. Schauberger P, Walker A (2025). openxlsx: Read, Write and Edit xlsx Files. R package version 4.2.8, https://github.com/ycphs/openxlsx, https://ycphs.github.io/openxlsx/index.html.

7. Gu Z (2022). “Complex Heatmap Visualization.” iMeta. doi:10.1002/imt2.43.

8. Gu Z, Eils R, Schlesner M (2016). “Complex heatmaps reveal patterns and correlations in multidimensional genomic data.” Bioinformatics. doi:10.1093/bioinformatics/btw313.

9. Wickham H, François R, Henry L, Müller K, Vaughan D (2025). dplyr: A Grammar of Data Manipulation. R package version 1.1.4, https://dplyr.tidyverse.org.

10. Wickham H, Vaughan D, Girlich M (2025). tidyr: Tidy Messy Data. R package version 1.3.1, https://tidyr.tidyverse.org.

11. Garnier, Simon, Ross, Noam, Rudis, Robert, Camargo, Pedro A, Sciaini, Marco, Scherer, Cédric (2024). viridis(Lite) - Colorblind-Friendly Color Maps for R. doi:10.5281/zenodo.4679423, viridis package version 0.6.5, https://sjmgarnier.github.io/viridis/.

12. Dunnington D (2025). ggspatial: Spatial Data Framework for ggplot2. R package version 1.1.10, https://paleolimbot.github.io/ggspatial/.

13. Wickham H, Hester J, Bryan J (2025). readr: Read Rectangular Text Data. R package version 2.1.5.9000, https://github.com/tidyverse/readr.

14. Pebesma, E., & Bivand, R. (2023). Spatial Data Science: With applications in R. Chapman and Hall/CRC.

15. Pebesma, E. (2018). Simple Features for R: Standardized Support for Spatial Vector Data. The R Journal, 10(1), 439–446.

16. Fotheringham, A. S., Brunsdon, C., & Charlton, M. (2002). Geographically weighted regression: The analysis of spatially varying relationships. John Wiley & Sons.

17. Massicotte P, South A (2025). rnaturalearth: World Map Data from Natural Earth. R package version 1.1.0.9000, https://docs.ropensci.org/rnaturalearth/.

18. Garrett Grolemund, Hadley Wickham (2011). Dates and Times Made Easy with lubridate. Journal of Statistical Software, 40(3), 1-25. URL https://www.jstatsoft.org/v40/i03/.

19. Wickham H, Henry L (2025). purrr: Functional Programming Tools. R package version 1.1.0, https://purrr.tidyverse.org/.

20. Auguie, B., & Antonov, A. (2010). gridExtra (Version 2.3) [R package].
